# Single-shot multiparametric MRI for separating *T_2_* effects from dynamic glucose-enhanced contrast

**DOI:** 10.7150/thno.116483

**Published:** 2025-08-30

**Authors:** Junxian Jin, Haizhen Ding, Zhekai Chen, Yuan Huang, Hongmin Chen, Zhong Chen, Lin Chen

**Affiliations:** 1 Department of Electronic Science, Fujian Provincial Key Laboratory of Plasma and Magnetic Resonance, School of Electronic Science and Engineering, National Model Microelectronics College, Xiamen University, Xiamen, China.; 2 State Key Laboratory of Vaccines for Infectious Diseases, Xiang An Biomedicine Laboratory, National Innovation Platform for Industry-Education Integration in Vaccine Research, School of Public Health, Xiamen University, Xiamen, China.

**Keywords:** glucose uptake, dynamic glucose enhanced, chemical exchange saturation transfer, multiparametric, single-shot

## Abstract

**Background:** Glucose is a central substrate in cellular metabolism and serves as a non-invasive biomarker for pathological processes. Dynamic glucose-enhanced (DGE) MRI based on chemical exchange saturation transfer (CEST) offers a promising tool for mapping glucose uptake, but its quantification is confounded by glucose-induced changes in *T_2_* relaxation in addition to glucose concentration.

**Methods:** We developed a single-shot multiparametric CEST (MP-CEST) MRI sequence based on multi-echo spatiotemporal encoding (SPEN), enabling the simultaneous acquisition of *T_2_* and saturation-weighted proton density (PD) maps within a single scan. To correct for *T_2_*-related confounding effects in glucoCEST quantification, a two-step correction strategy was employed. First, the saturation-weighted PD maps, which mitigate *T_2_*-dependent signal attenuation during image acquisition, were used to reconstruct the Z-spectrum, thereby providing a more accurate representation of the true saturation signal amplitude. Second, calibration curves derived from Bloch-McConnell simulations were applied in combination with the simultaneously acquired *T_2_* maps to compensate for spillover effects in the Z-spectrum, thereby improving glucose-specific CEST contrast. The full framework was validated through phantom experiments and *in vivo* studies in rat brain and tumor xenograft models. Quantitative performance was evaluated by computing the Pearson correlation between DGE signals and *T_2_* values before and after correction, as well as by comparing fitted *T_2_* and PD values with reference maps.

**Results:** Phantom experiments demonstrated high accuracy in PD and *T_2_* quantification (R^2^ > 0.99). *In vivo* studies in rat brain and tumor xenografts showed that the proposed correction method significantly reduced the correlation between DGE signals and *T_2_* values, improving the specificity of glucose-related contrast. In addition, *T_2_* maps provided complementary structural and physiological information relevant to tumor heterogeneity and tissue microstructure.

**Conclusions:** The proposed MP-CEST approach improves the robustness and accuracy of DGE quantification, offering a more comprehensive metabolic imaging framework applicable to both oncological and neurological research.

## Introduction

Glucose is the primary source of energy in most organisms 1, and abnormal glucose metabolism is a hallmark of numerous diseases, such as cancer 2, 3, diabetes 4, 5, and Alzheimer's disease 6, 7. Monitoring glucose dynamics is not only critical for maintaining metabolic homeostasis and understanding physiological processes but also serves as a key biomarker for disease diagnosis, progression tracking, and therapeutic response assessment 8, 9. Given its clinical significance, developing sensitive and non-invasive glucose imaging techniques is valuable for improving the detection and management of glucose-related disorders 10.

For several decades, glucose uptake and metabolism have been assessed non-invasively using [18F]-fluorodeoxyglucose positron emission tomography (FDG-PET) 11. Despite its great detection sensitivity, the high cost of FDG-PET limits its widespread clinical application. In addition, the use of a radioactive tracer is not ideal for repeated measurements, especially when combined with CT for anatomical referencing 12. Alternative methods for assessing glucose uptake and utilization include ^13^C, ^2^H and ^1^H *in vivo* magnetic resonance spectroscopy (MRS) 13-15, along with a novel deuterium metabolic imaging approach 16. Although MRS techniques have been successful in studying basic metabolic processes during glucose infusion, their limited detection sensitivity and spatial resolution pose challenges for clinical implementation.

Recently, glucose chemical exchange saturation transfer (glucoCEST) MRI has been well-established for detecting unlabeled glucose at physiologically relevant concentrations by exploiting the interaction between hydroxyl protons and water 1, 17, 18. The time-resolved variant, known as Dynamic Glucose-Enhanced (DGE) MRI, measures dynamic changes in MR signals induced by variations in glucose concentration following administration. This approach was developed to study glucose dynamics with high temporal resolution by omitting the time-consuming acquisition of full spectra 19, 20. As research progressed, it became evident that fluctuations in glucose concentration also modulate tissue *T_2_*, as reported in previous studies 21. This newly recognized *T_2_*-dependent effect introduces a confounding influence on the interpretation of DGE signals via two primary mechanisms. First, the glucoCEST resonance lies in close spectral proximity to the bulk water signal (~1 ppm), making it highly vulnerable to spillover artifacts. The extent of this spillover is known to depend on tissue *T_2_* relaxation, implying that variations in *T_2_* can indirectly alter the observed glucoCEST contrast by modulating the degree of signal contamination 22, 23. Second, *T_2_* decay during image acquisition can influence the measured CEST signal 24; thus, variations in *T_2_* may induce glucoCEST signal changes that reflect glucose concentration in a confounded or more complex manner 21. Despite the potential for these *T_2_*-related effects to bias both the sensitivity and specificity of DGE measurements, they have received limited systematic investigation. To date, few studies have quantitatively assessed the impact of *T_2_* variability or developed correction strategies to mitigate its confounding influence, leaving a critical methodological gap in the accurate interpretation of glucose-enhanced MRI data.

In this study, we address the confounding impact of *T_2_* variability on DGE signal quantification by proposing a novel single-shot multiparametric CEST (MP-CEST) technique based on multi-echo spatiotemporal encoding (SPEN). This approach enables the simultaneous acquisition of *T_2_* and saturation-weighted proton density (PD) maps through numerical fitting of multiple *T_2_*-weighted images acquired at different echo times 25, 26. By leveraging the saturation-weighted PD signal for DGE quantification, our method minimizes *T_2_* decay-related modulation effects during image readout. In addition, we derived *T_2_* calibration curves based on Bloch-McConnell simulations and *T_2_* maps to retrospectively correct for *T_2_*-induced changes occurring during CEST saturation. The feasibility and efficacy of this correction strategy were validated through both phantom experiments and *in vivo* imaging in rat brain and subcutaneous tumor xenograft models. Statistical evaluation demonstrated the potential of the proposed MP-CEST approach to improve the reliability of DGE measurements under varying *T_2_* conditions.

## Methods

### MP-CEST MRI sequence

**Figure 1:**
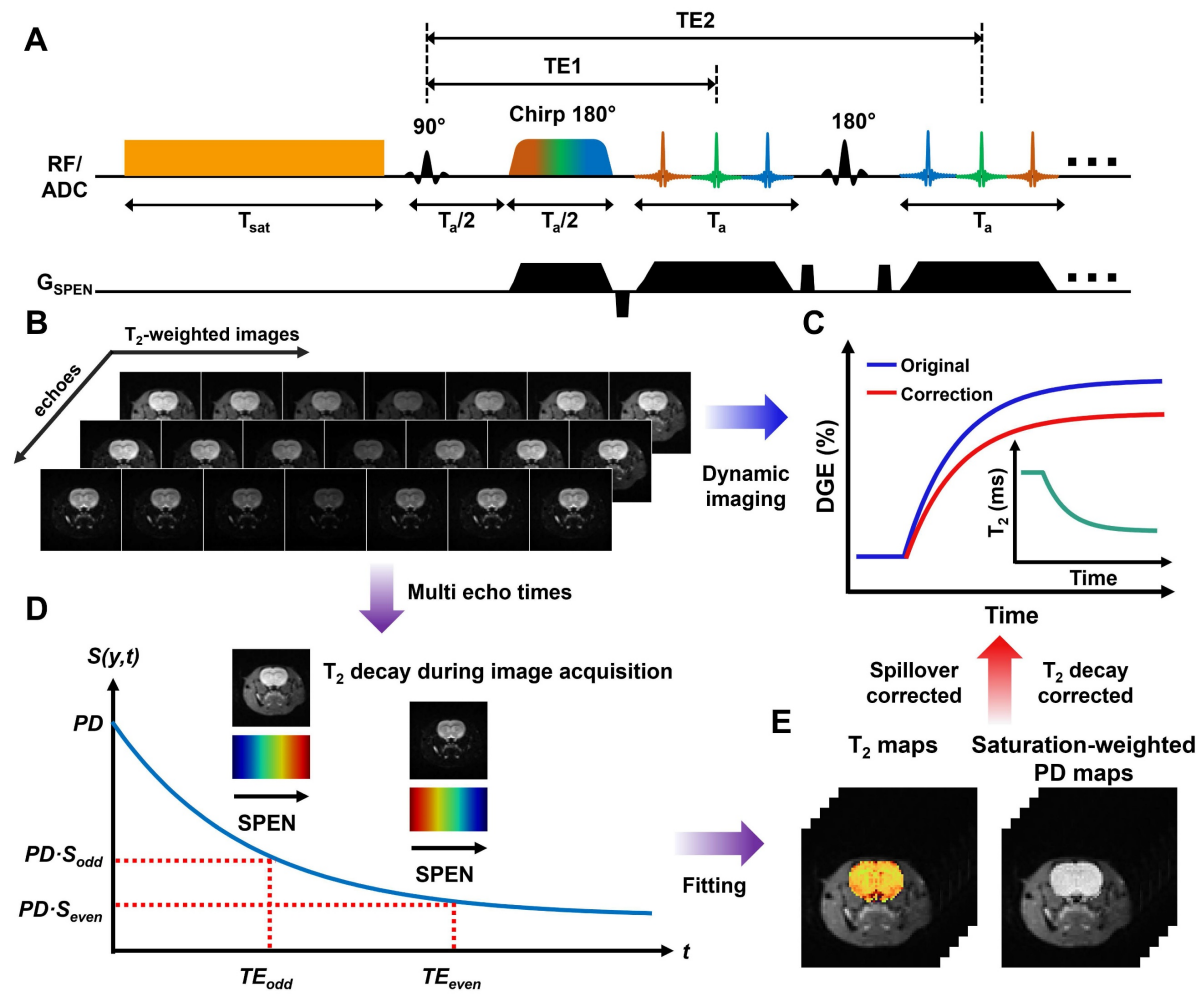
**Schematic overview of single-shot multiparametric CEST (MP-CEST) MRI framework. (A)** Timing diagram of the MP-CEST sequence. A long rectangular saturation pulse with duration t_sat_ and amplitude B_1_, tuned to a frequency offset Δω from the water resonance, is applied prior to data acquisition using a hybrid SPEN readout followed by a multi-echo echo train. **(B)** Representative *T_2_*-weighted images acquired at multiple echo times and frequency offsets in an in vivo rat brain. **(C)** DGE signal curves before and after *T_2_* correction, along with a conceptual diagram of dynamic *T_2_* changes over time. **(D)** Illustration of signal decay over time across multiple echo times, showing *T_2_*-dependent signal attenuation. **(E)** Simultaneous *T_2_* and saturation-weighted PD maps obtained using a model-based fitting approach applied to the multi-echo SPEN data.

The MP-CEST MRI sequence proposed in this study is illustrated in **Figure 1A**. It involves a long rectangular pulse to induce saturation transfer effects, consisting of a presaturation period with duration t_sat_ and an irradiation field with amplitude B_1_, tuned to a frequency offset ∆ω from the water proton resonance. The CEST image was acquired using SPEN imaging module with multi-echo train acquisitions. The encoding phase following the application of chirp pulses in SPEN MRI can be expressed as 25, 26:


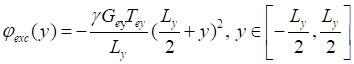

(1)

where γ represents the gyromagnetic ratio, G_ey_ and T_ey_ are the amplitude and duration of the encoding gradient along the y dimension, respectively. L_y_ is FOV along the y dimension. The phase variation during the acquisition period t can be expressed as


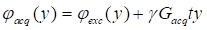

(2)

where G_acq_ is the acquisition gradient, for simplicity, we assume G_acq_ to be constant.

A multi-echo train is generated by interleaving 180° sinc pulses with signal acquisition modules, and the phase modulation of the odd and even echo trains can be described as follows:


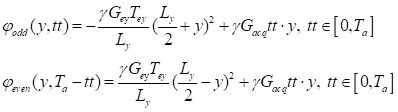

(3)

where T_a_ represents the acquisition period for each echo train, and tt is the evolution time during each decoding segment of the echo train. Representative multi-echo images acquired at different frequency offsets in an *in vivo* rat brain using this sequence are shown in **Figure 1B**. Based on the characteristics of the SPEN approach 27-29, the main contribution to the acquired signal energy comes from the spin density around the stationary point of the quadratic phase profile. Therefore, each sampled signal is modulated by both spatial encoding and *T_2_* decay, as described below:


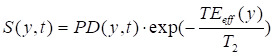

(4)

where TE_eff_ refers to the nominal echo time. Based on Eq. 4 and TE_eff_, voxel-wise mono-exponential fitting of the multi-echo images was performed to estimate both *T_2_* and saturated-weighted PD. The complete data processing workflow and a representative fitting result are illustrated in Supplementary Section 1 (**Figures S1-2**). The characteristic signal modulation behavior of SPEN MRI enables simultaneous quantification of saturation-weighted PD and *T_2_* relaxation through numerical fitting of multiple *T_2_*-weighted images acquired at different echo times 25, 26, as illustrated in **Figure 1D-E**.

### Theoretical Derivation

According to previous studies 30, 31, the normalized saturation signal *S* can be well described by the R_1ρ_ relaxation theory, which is formulated as follows 32, 33:


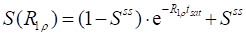

(5)


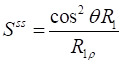

(6)

where S_ss_ refers to the Z-spectral intensity at steady-state, R_1_ is the longitudinal relaxation rate of water, t_sat_ is the saturation time, and θ = tan^-1^ω_1_/∆ is the tilt angle of the effective magnetization with respect to the Z-axis induced by a saturation pulse with nutation frequency ω_1_ at an offset ∆, R_1ρ_ represents the water relaxation rate under a saturation pulse, incorporating both the effective water relaxation (R_eff_) and an apparent saturation transfer-related term (R_ST_) reflecting all exchange processes in tissue 22:


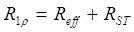

(7)

where R_eff_ = cos^2^θ∙R_1_ + sin^2^θ∙R_2_ represents the effective longitudinal relaxation rate of water in the rotating frame in the absence of additional solute or exchange components. Based on the R_1ρ_ theory, the contributions from multiple CEST effects can be linearly combined, as R_1ρ_ exhibits an approximately additive response to different exchanging pools 33, 34. In this study, we employed a two-pool model comprising a glucose pool and a background pool representing all other exchanging protons to analyze the CEST signal, as described below:


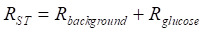

(8)

By combining Eqs. 6 and 7, the S^ss^ can be derived as follows:


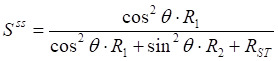

(9)

For *in vivo* glucoCEST experiments, the glucose pool resonates close to water, and the effect of R₂ cannot be neglected. When R_2_ is much greater than cos^2^θ∙R_1_ + R_ST_, (where R_2_ > 14 s^-1^ within the physiological range and cos^2^θ∙R_1_ + R_ST_ is approximately 1 s⁻¹), the S^ss^ in Eq. 6 can be approximated as follows:


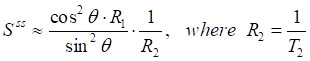

(10)

The MTR_asym_, which isolates the glucoCEST effect by subtracting the background signal, is defined as:


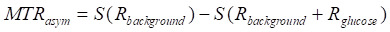

(11)

For *in vivo* glucoCEST experiments at a B_1_ of 2 μT, the glucose-related relaxation rate (*R*_glucose_) is much smaller than the background relaxation rate (*R*_background_) due to strong MTC effects and spillover effect (i.e., *R*_glucose_ ≪ *R*_background_). Under this condition, the MTR_asym_ in Eq. 11 can be approximated as follows:


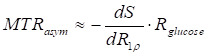

(12)

where dS/dR_1ρ_ can be calculated from Eq. 5, and is given by:


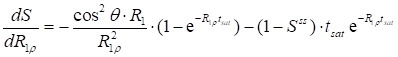

(13)

By combining Eqs. 12 and 13, the MTR_asym_ can be derived as follows:


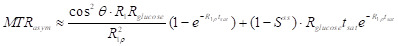

(14)

Under steady-state saturation, the MTR_asym_ can be simplified as follows 30:


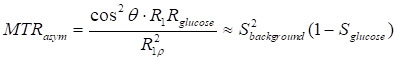

(15)

where *S*_background_ and *S*_glucose_ represent the normalized saturation signal at steady-state when only the background or glucose pool is present, respectively. This indicates that the MTR_asym_ can be significantly attenuated by a factor of 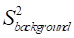
in the presence of a strong background signal, a phenomenon referred to as the “scaled-down effect” in our previous studies 30, 31.

In this study, we propose a correction algorithm to remove the *T_2_* effect, thereby improving the correlation between DGE signal changes and glucose concentration toward a more linear relationship. According to Eq. 10, the relationship between *S*_background_ and the change in *T_2_* can be expressed as follows:


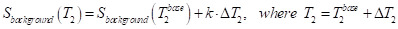

(16)

where 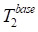
denotes the baseline transverse relaxation time, ∆*T*_2_ represents the change of transverse relaxation time during the DGE experiment, *k* characterizes the linear sensitivity of the background signal to *T*_2_ variations. Therefore, a linear correction model was applied to mitigate *T_2_*-induced variations in the background signal of the single-offset DGE measurement.

From Eq. 15, the background signal induces a scaled-down effect on the observed MTR_asym_. The contribution to the observed MTR_asym_ due to background fluctuations can be formulated as:


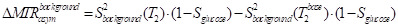

(17)

By combining Eqs. 16 and 17, the 
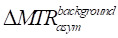
can be formulated as:


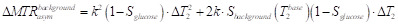

(18)

Eq. 18 represents the analytical expression for the background-induced variation in MTR_asym_ as a function of Δ*T_2_*. To facilitate fitting and correction, this relationship was approximated by a second-order polynomial, as described below:


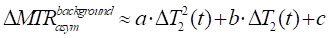

(19)

where *a* and *b* are the coefficients of a second-order polynomial that describe the relationship between Δ*T_2_* and the background-induced contribution to observed MTR_asym_ change, the constant term *c* is included to account for residual baseline shifts and other unmodeled systematic variations that may arise in experimental data. The corrected MTR_asym_ signal was obtained by subtracting the fitted background contribution 
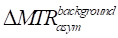
from the original MTR_asym_ signal.

Correction parameters (*k*, *a*, *b*, and *c*) were derived from Bloch-McConnell simulations based on experimental and literature-informed parameters for the phantom 21, *in vivo* mouse brain 35, 36, and tumor 37, 38, respectively. For the phantom experiment, the background consisted solely of water, whereas in the* in vivo* setting, an additional magnetization transfer contrast (MTC) component was included, as detailed in **Table S1**. Validation of the model's robustness and generalizability is presented in Supplementary Section 2 (**Figures S3-6**). All simulations and voxel-wise fittings were implemented using custom MATLAB scripts (R2019b, MathWorks), with corrections applied according to Eqs. 16 and 19. A schematic overview of the DGE signal dynamics before and after *T_2_* correction, along with a conceptual illustration of temporal *T_2_* changes, is presented in **Figure 1C**.

### Numerical simulation validation

Numerical simulations were conducted to evaluate the effectiveness of spillover correction under varying *T_2_* conditions. According to the previous study 21, changes in glucose concentration will alter the tissue *T_2_* value, which can be described as follows:


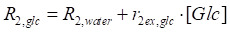

(20)

where R_2,water_ refers to the tissue R_2_ value without glucose change and R_2,glc_ refers to the tissue R₂ value after a change in glucose concentration [Glc]. r_2ex, glc_ represents the transverse relaxivity of glucose, which was set to 0.053 s^-1^ mM^-1^ under 7 T conditions, based on the previous study. Z-spectra were simulated to model glucose exchangeable protons at 1.2 ppm, with concentrations ranging from 0 to 500 mM in 10 mM increments. Frequency offsets were sampled from -5 to 5 ppm in 0.1 ppm steps. Saturation power and duration were set to 2 µT and 2 s, respectively. Additional simulation parameters are detailed in Supplementary **Table S2**.

### Phantom experiments

D-glucose solutions (10-310 mM; 10, 50, 100, 150, 230, and 310 mM) were prepared in phosphate-buffered saline (PBS) containing 2 mM Gd-DTPA, with pH adjusted to 7.2, and experiments were conducted at 22 °C. Experiments were conducted on a 7 T Varian MRI system (Varian Associates, Palo Alto, CA) equipped with a horizontal-bore Magnex magnet and 10 cm-bore imaging gradient coils. The MP-CEST sequence was implemented in the VNMRJ 4.0 environment. Imaging parameters were: saturation power = 2 µT, saturation duration = 2000 ms, FOV = 50 × 50 mm^2^, slice thickness = 2 mm, chirp time-bandwidth product = 120, 5-echo train with 25 ms echo spacing, and TR = 5000 ms. Frequency offsets ranged from -5 to 5 ppm with 0.2 ppm steps. Reference *T_2_* and PD maps were acquired using spin-echo imaging with multiple echo times (TE = 12, 20, 32, 50, 70, 100, 150 ms; TR = 2000 ms), and the total scan time was 22 min 24 s. Details of the optimization process for echo spacing and the number of echoes are provided in Supplementary Section 4 (**Figure S7 and Table S3**).

### Animal preparations

All animal procedures were approved by the Animal Experimental Center of Xiamen University. In this study, six female Sprague-Dawley (SD) rats (aged 9-11 weeks, weighing 190-220 g) and three female BALB/c mice were used. All animals were purchased from Shanghai SLAC Laboratory Animal Co., Ltd. (Shanghai, China).

Tumor-bearing mice were established by subcutaneous injection of 5 × 10^6^ 4T1 cells into the right hind limb of 6-week-old BALB/c mice (∼20 g). MRI was performed when tumor volumes reached ~100 mm³. Prior to imaging, animals were anesthetized with 4% isoflurane (rats) or 2% isoflurane (mice) for induction, followed by maintenance at 2% and 1%, respectively, during scanning. A tail vein catheter was placed for intravenous glucose administration. Dynamic imaging was performed continuously, starting before and continuing through the 1-minute intravenous injection of filtered D-glucose solution (50% w/w, 0.5 g/mL, ~2.78 M; 1.5 mL for rats; 0.1 mL for mice). Several baseline images were acquired prior to injection and used as references for subsequent normalization of the DGE signal.

### *In vivo* experiments

*In vivo* imaging was performed on a 7 T Varian MRI scanner (Varian Associates, Palo Alto, CA) equipped with a horizontal-bore Magnex magnet and 10 cm-bore gradient coils. B_0_ field homogeneity over the rat brain was optimized using field mapping and second-order shimming before DGE acquisition. The MP-CEST sequence was applied with the following parameters: saturation power = 2 µT, saturation duration = 2000 ms, FOV = 45 × 45 mm^2^, slice thickness = 2 mm, chirp time-bandwidth product = 120, 5-echo train with 25 ms echo spacing, and TR = 5000 ms. For dynamic DGE imaging, CEST data were acquired at ±2.0, ±1.5, ±1.2, ±0.9, and 0 ppm offsets relative to water. Signal intensity at 1.2 ppm was extracted for DGE quantification, yielding a temporal resolution of 45 s per image pair. A total of 2700 images were acquired over 46 minutes. Each dynamic repetition included five echoes across nine frequency offsets, resulting in 45 echo-offset combinations per time point. Sixty dynamic repetitions were acquired, yielding 60 time points for DGE analysis. Every set of five echoes was used to generate *T_2_* and saturation-weighted PD maps. Dynamic scanning began with 10 baseline scans over 7 min 30 s, followed by a 1-minute intravenous injection of 50% D-glucose and continued acquisition for 37 min 30 s post-injection.

### MRS

To verify glucose uptake, localized proton MRS was acquired twice: once immediately before glucose injection and once following the completion of the MP-CEST scan. This sequential protocol ensured that the MRS measurements reflected pre- and post-infusion metabolite levels without interrupting the dynamic glucoCEST imaging. All MRS scans were performed using a point-resolved spectroscopy (PRESS) sequence with outer volume suppression (OVS). Water suppression was achieved using the variable power RF pulses and optimized relaxation delays (VAPOR) technique 39. Second-order localized shimming was performed using B_0_ field mapping prior to each acquisition. The MRS protocol used the following parameters: TR = 2000 ms, TE_1_ = 7.5 ms, TE_2_ = 6 ms, 4096 complex points, NEX = 128, and a total acquisition time of 4 min 16 s. For *in vivo* MRS acquisition, a single voxel (4 × 4 × 4 mm³) was placed in the posterior thalamic region of the rat brain and the dorsal region of the subcutaneous tumor, respectively. These locations were selected to ensure inclusion of relatively large and homogeneous tissue areas, minimizing partial volume effects and susceptibility-induced distortions.

### Data analysis

A signal normalization process was applied to the Z spectrum using saturation-to-baseline ratio normalization, defined as the ratio of the water signal during saturation to the unsaturated baseline signal:


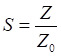

(21)

where S is the normalized image intensity, Z refers to the saturated signal obtained after applying the saturation pulse (e.g., saturation-weighted PD or *T_2_*-weighted images), while Z₀ is the baseline signal acquired without saturation and is used for normalization.

DGE MRI is defined as the dynamic changes in MR signals induced by variations in glucose concentration following administration. For single-offset DGE quantification, which measures the temporal change of the CEST signal at 1.2 ppm, normalization was applied using the pre-injection baseline signal *S*, as formulated below:


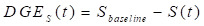

(22)

where S_baseline_ denotes the average of all pre-injection *T_2_*-weighted CEST images or PD maps, and S(t) is the corresponding signal at time t.

For MTR_asym_ DGE signal quantification, the signal was defined as the temporal difference at 1.2 ppm in the MTR_asym_ spectrum relative to the pre-injection baseline:


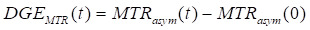

(23)

To correct for motion between CEST images, image registration was performed using the Medical Imaging Registration Toolbox 40. The unsaturated image was used as the reference target, and all other CEST images were aligned to it. The registration parameters were as follows: similarity measure set to residual complexity, three hierarchical resolution levels, mesh window size of 8, regularization weight of 0.005, maximum of 200 iterations, tolerance of 1 × 10⁻⁵, and annealing rate of 0.8. Regions of the rat brain were then automatically segmented 41 based on the registered standard rat brain atlas (http://atlas.brain-map.org/).

For LCModel analysis, the total creatine (tCr) concentration was used as an internal reference to quantify D-glucose uptake 42. Specifically, the glucose concentration change (Δ[Glc]) following D-glucose administration was estimated using the area ratio between the difference spectrum of the D-glucose peaks (3.4-4.0 ppm) and the tCr methyl peak (3.0 ppm) in the pre-injection reference spectrum (RGlc/tCr), according to the following equation:


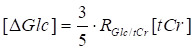

(24)

where 3/5 represents the proton ratio between the methyl peak of total creatine (tCr) at 3.0 ppm (three protons) and the combined D-glucose peaks between 3.4 and 4.0 ppm (five protons).

### Statistical analysis

All statistical analyses were performed using MATLAB (R2019b, MathWorks). Quantitative data are reported as mean ± standard deviation (SD). Group comparisons were assessed using unpaired two-tailed Student's t-tests or one-way analysis of variance (ANOVA), as appropriate. A P-value < 0.05 was considered statistically significant. Pearson correlation coefficients (R) and corresponding P-values were calculated to assess the linear association between DGE signals and *T_2_* values before and after correction.

## Results

### Validation of MP-CEST-derived PD and *T_2_* mapping

**Figure 2:**
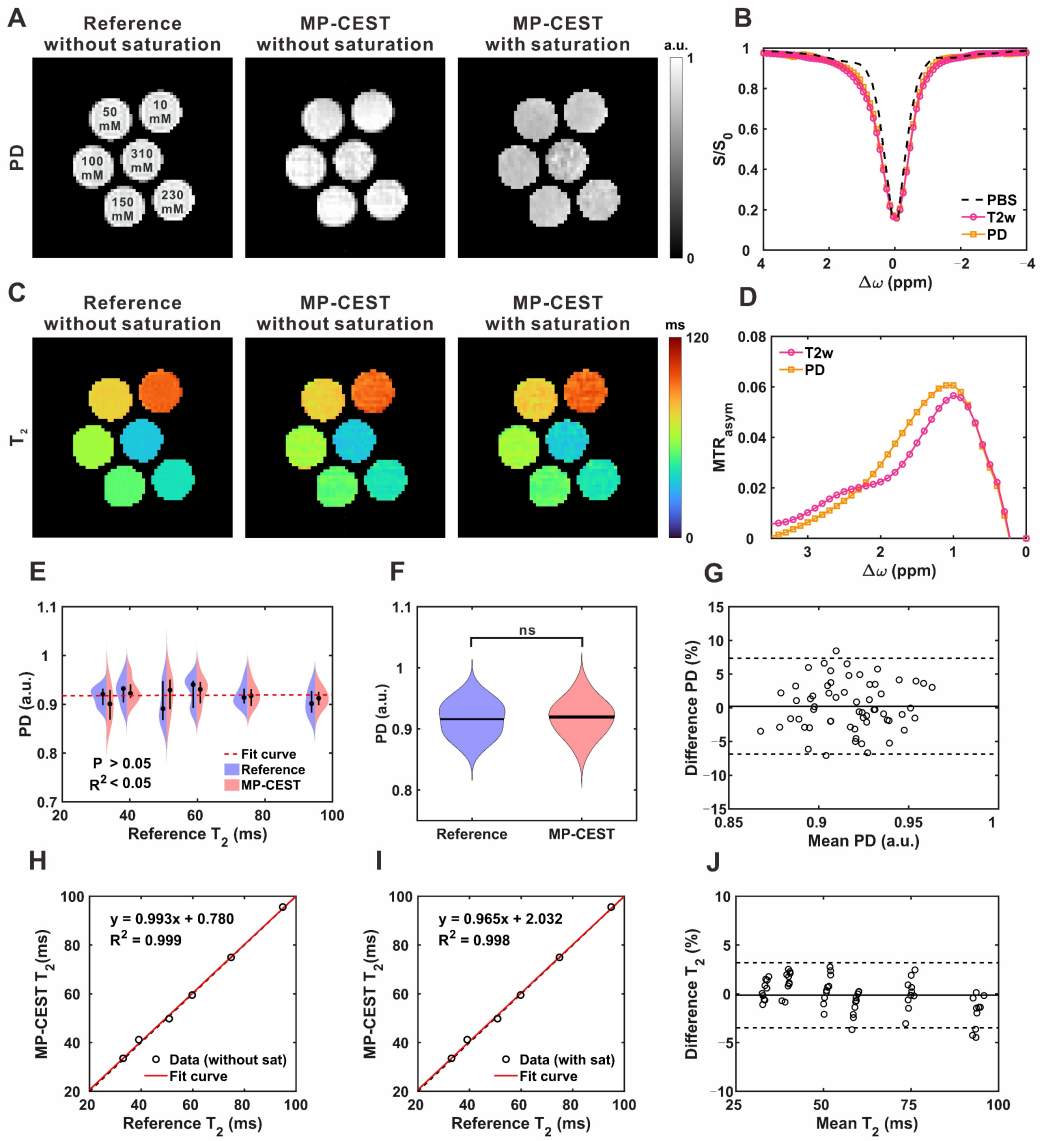
**GlucoCEST imaging of glucose phantoms at 7 T using the proposed MP-CEST method**. **(A)** PD maps obtained from the reference and proposed methods, both with and without saturation. **(B)** Z-spectra derived from *T_2_*-weighted images and saturation-weighted PD images at a glucose concentration of 50 mM. **(C)** *T_2_* maps corresponding to the conditions in (A). **(D)** Corresponding MTR_asym_ plots calculated from the respective Z-spectra. **(E)** Violin plots comparing PD values versus *T_2_* values between the reference and proposed methods. **(F)** Violin plots showing direct PD value comparisons between the two methods. **(G)** Bland-Altman plot assessing agreement in PD values between the proposed and reference methods. **(H, I)** Scatter plots of quantitative *T_2_* values from the six glucose tubes, without (H) and with (I) saturation. **(J)** Bland-Altman plot comparing *T_2_* values from the proposed and reference methods.

**Figure 2** summarizes the validation of MP-CEST-derived PD and *T_2_* mapping against reference measurements in phantom experiments. PD and *T_2_* maps acquired using MP-CEST, with and without saturation, along with reference maps, are shown in **Figure 2A and 2C**; additional maps are provided in **Figure S8**. Violin plots (**Figure 2E-F**) show that, in the absence of saturation, there was no significant difference between MP-CEST-derived PD values and the reference PD measurements (P = 0.639). Additionally, no correlation was observed between PD and *T_2_* values (R² < 0.001). In contrast, *T_2_* values exhibited strong linear agreement with reference measurements (R^2^ = 0.999 without saturation; R^2^ = 0.998 with saturation; **Figure 2H-I**). Bland-Altman analysis (**Figure 2G and 2H**) further confirmed the agreement, with MP-CEST showing a mean bias of -0.24% for PD and +0.13% for *T_2_* compared to the reference. These results indicate that saturation leads to a frequency-dependent reduction in PD values. For example, the reduction was 2.61% ± 1.15% at 3.0 ppm (P = 0.028, effect size = 0.408) and 20.81% ± 4.36% at 1.2 ppm (P < 0.001, effect size = 3.117). In contrast, *T_2_* measurements were minimally affected, suggesting that the observed changes primarily reflect alterations in proton density. GlucoCEST signals originating from hydroxyl protons were observed as asymmetry in the 0-2 ppm range in the tube containing 50 mM glucose (**Figure 2B**), and became more pronounced in the corresponding MTR_asym_ plot (**Figure 2D**). The Z-spectrum derived from saturation-weighted PD images exhibited a significantly narrower spectral peak compared to that from *T_2_*-weighted images, with a full width at half maximum (FWHM) of 0.947 ± 0.074 ppm versus 0.973 ± 0.075 ppm (P = 0.028). Similar results were observed for the tube containing 10 mM glucose (**Figure S9**). This narrowing enhances the specificity for glucose-related exchangeable protons and indicates effective mitigation of *T_2_*-dependent signal broadening during acquisition.

### Evaluation of *T_2_* effects on CEST metrics and correction performance

**Figure 3:**
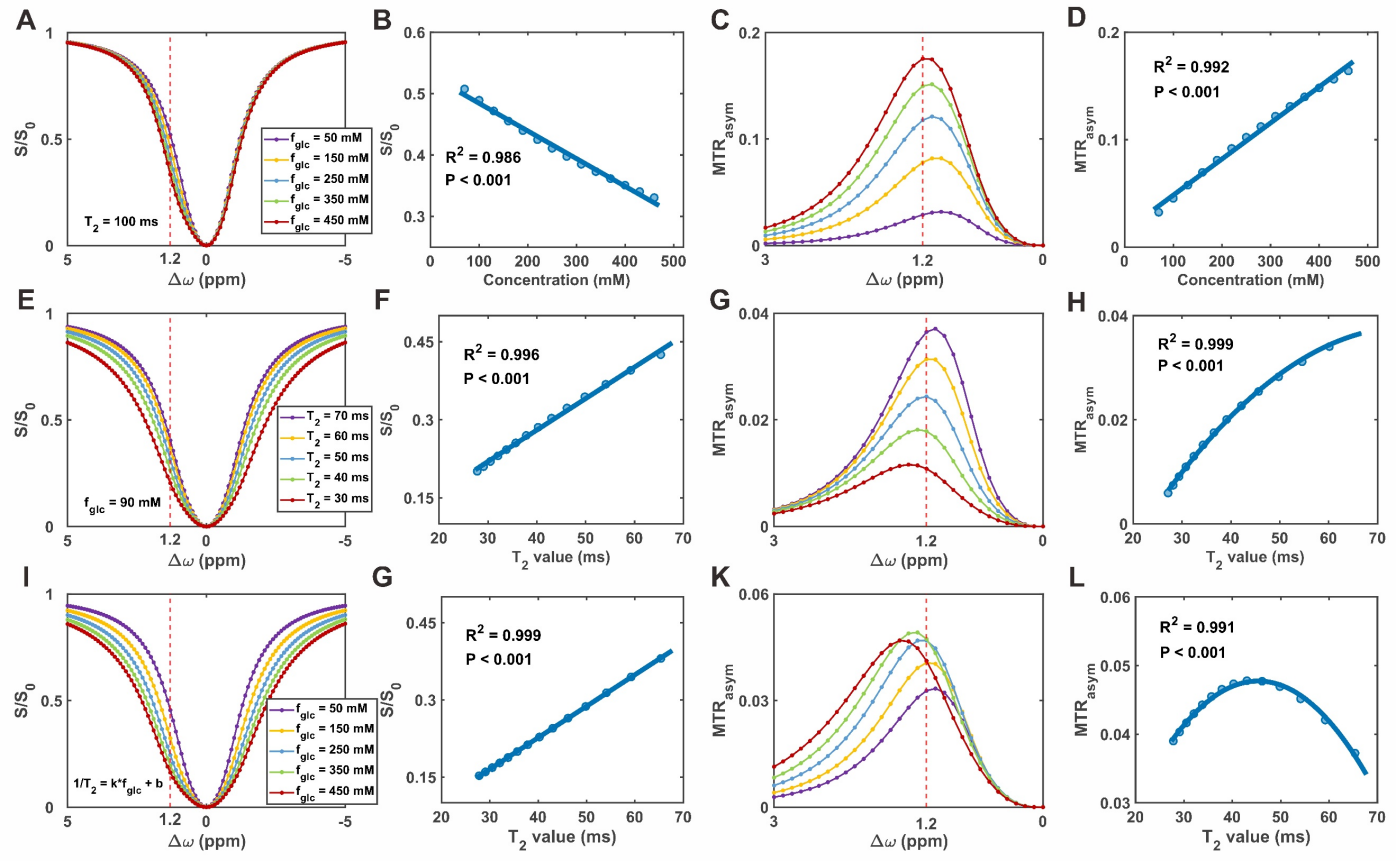
**Simulation of glucoCEST signal under varying glucose concentrations and *T_2_* relaxation times. (A)** Simulated Z-spectra at different glucose concentrations. **(B)** Correlation between glucose concentration and normalized saturation signal (S/S_0_). **(C)** MTR_asym_ curves corresponding to (A). **(D)** Correlation between glucose concentration and MTR_asym_. **(E)** Simulated Z-spectra at varying *T_2_* relaxation times. **(F)** Correlation between *T_2_* and S/S_0_. **(G)** MTR_asym_ curves corresponding to (E). **(H)** Correlation between *T_2_* and MTR_asym_. **(I)** Z-spectra under combined variations of *T_2_* and glucose concentration. **(J)** Correlation between combined *T_2_*-glucose variations and S/S_0_. **(K)** MTR_asym_ under the same combined conditions. **(L)** Correlation between combined *T_2_*-glucose variations and MTR_asym_.

**Figure 3A-D** presents the simulation results obtained under varying glucose concentrations with a fixed *T_2_* value. As glucose concentration increased, the normalized signal (S/S_0_) decreased near the 1.2 ppm offset, while MTR_asym_ increased (**Figure 3A and 3C**). These effects were quantified by fitting S/S_0_ and MTR_asym_ to glucose concentration (**Figure 3B and 3D**), revealing a linear negative correlation for S/S_0_ and a linear positive correlation for MTR_asym_. To evaluate the effect of *T_2_* relaxation on CEST metrics, simulations were conducted with varying *T_2_* values while keeping glucose concentration constant.

As *T_2_* increased, Z-spectrum intensities rose across the entire frequency offset range (-5 to +5 ppm), accompanied by an increase in MTR_asym_ (**Figure 3E and 3G**). Quantitative analysis demonstrated a linear positive relationship between *T_2_* and S/S_0_, while MTR_asym_ exhibited a second-order (quadratic) dependence on *T_2_* (**Figure 3F and 3H**). Under physiological conditions, increases in glucose concentration are typically associated with reductions in *T_2_* relaxation times. To account for this coupling, simulations were performed in which glucose concentration and the corresponding *T_2_* values were varied simultaneously. As shown in the Z-spectrum (**Figure 3I**), increasing glucose concentration, along with the associated decrease in *T_2_*, resulted in a cumulative effect that led to a further reduction in S/S_0_. In contrast, the effect on MTR_asym_ at 1.2 ppm was more complex, exhibiting a biphasic response characterized by an initial increase followed by a subsequent decrease (**Figure 3K**). To better characterize these trends, the relationships between *T_2_* and CEST metrics were further analyzed. As shown in **Figure 3G and 3L**, S/S_0_ increased linearly with *T_2_*, while MTR_asym_ exhibited a second-order (quadratic) dependence. These findings support the correction model, in which *T_2_* and glucose linearly affect S/S_0_, while their combined effect on MTR_asym_ follows a quadratic, scale-down behavior. Additional evaluations of which form of transverse relaxation is more suitable for correction are shown in **Figure S10**.

**Figure 4:**
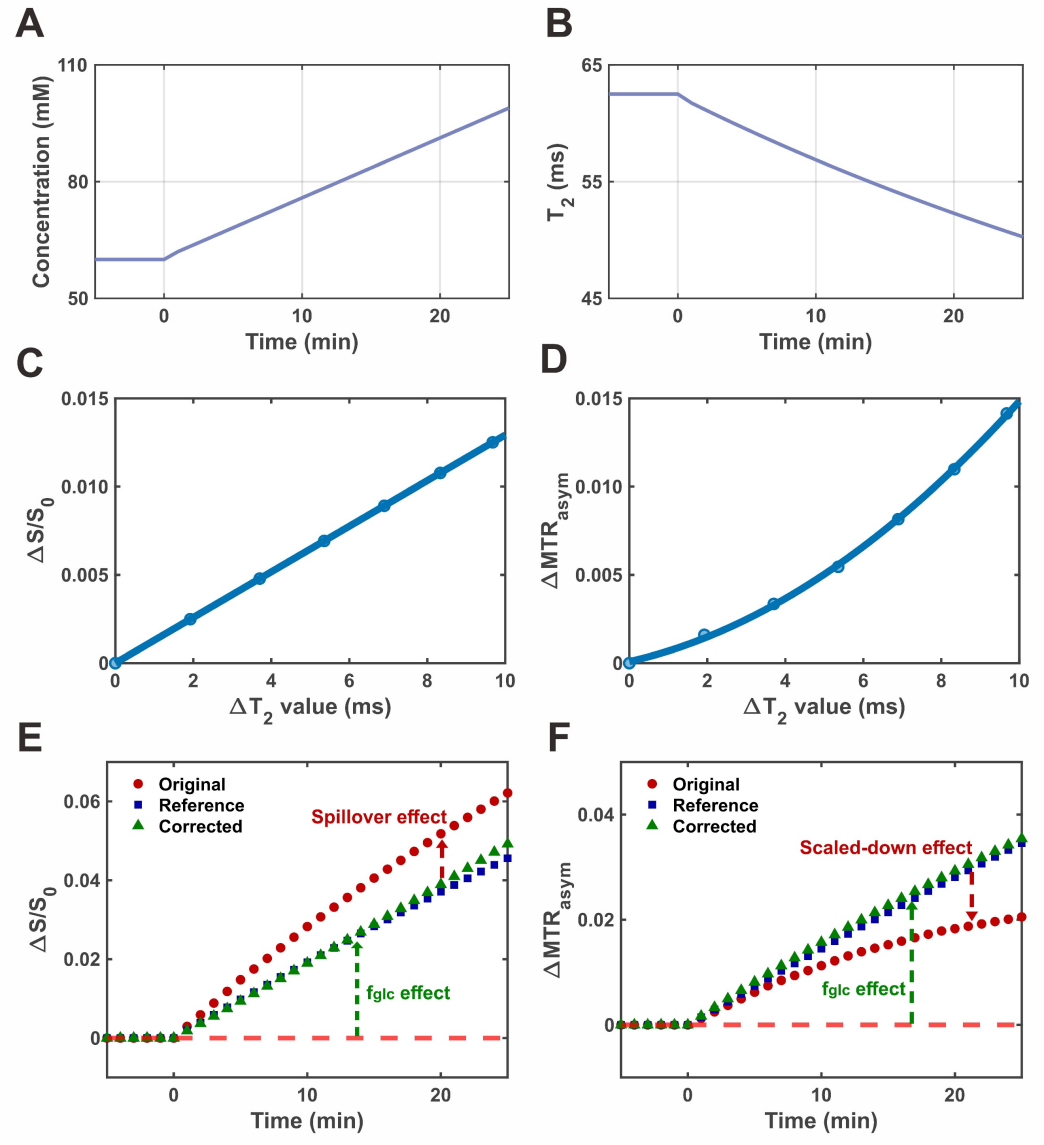
** Simulation of DGE CEST imaging and *T_2_* correction. (A)** Simulated time-resolved curve of glucose concentration during glucose infusion. **(B)** Corresponding dynamic *T_2_* relaxation curve derived from the glucose concentration profile. **(C)** Calibration curve showing the dependence of ΔS/S_0_ on *T_2_* variation. **(D)** Calibration curve showing the dependence of ΔMTR_asym_ on *T_2_* variation. **(E)** Time-resolved single-shot DGE signal (ΔS/S_0_) from original, corrected, and reference data. **(F)** Time-resolved MTR_asym_-based DGE signal from original, corrected, and reference data.

To evaluate the performance of the proposed correction strategy under dynamic conditions, a simulated DGE experiment was conducted incorporating time-dependent changes in both glucose concentration and *T_2_* relaxation. **Figure 4** summarizes the simulation framework and correction outcomes. The temporal evolution of glucose concentration and corresponding *T_2_* values, mimicking physiological uptake dynamics, is shown in **Figure 4A-B**, respectively. As glucose concentration increased over time, *T_2_* decreased accordingly, consistent with previously reported *in vivo* observations. Based on the modeled relationships between Δ*T_2_* and both S/S_0_ and MTR_asym_ (Eqs. 16 and 19), correction curves were generated to describe their respective *T_2_* dependencies (**Figure 4C-D**). The single-offset DGE signal displayed a linear dependence on *T_2_*, whereas MTR_asym_-based DGE followed a second-order (quadratic) trend. These *T_2_*-related effects introduced systematic bias in the uncorrected signals: decreasing *T_2_* led to an overestimation of the single-offset DGE signal and an underestimation of the MTR_asym_ DGE signal (**Figure 4E-F**). The application of the correction curves effectively compensated for these distortions. The corrected signals showed improved temporal alignment with the reference dynamics and exhibited enhanced correlation with glucose concentration.

### *In vivo* validation of *T_2_* correction in brain and tumor imaging

**Figure 5:**
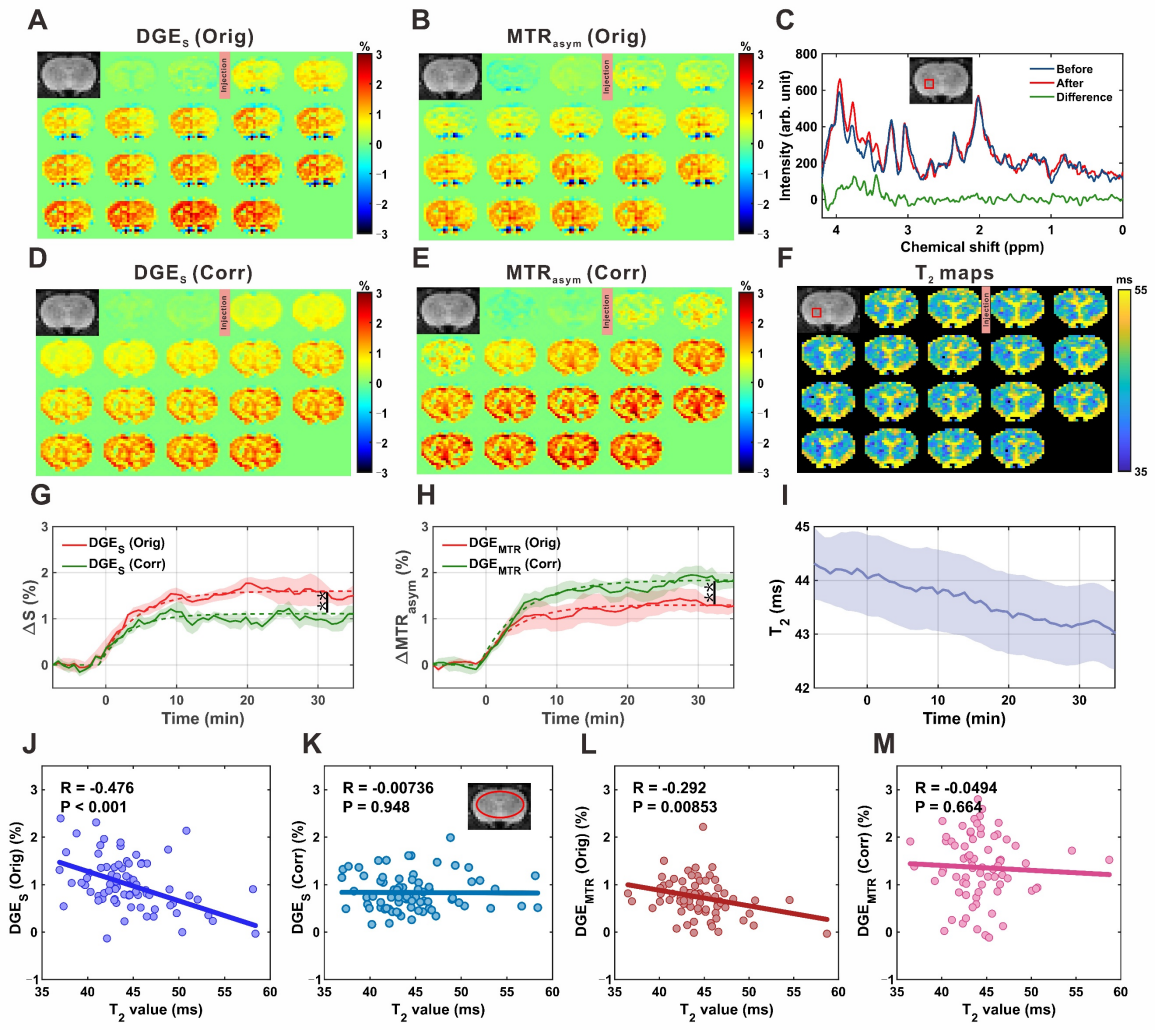
** MP-CEST MRI results of brain parenchyma following D-glucose infusion. (A, D)** Single-offset DGE images before and after glucose infusion, shown for original (A) and *T_2_*-corrected (D) data. **(B, E)** MTR_asym_-based DGE images for original (B) and corrected (E) datasets. **(C)** MRS spectrum showing glucose-enhanced signals between 3 and 4 ppm after infusion. **(F)** Dynamic *T_2_* maps before and after glucose infusion, illustrating *T_2_* relaxation changes. **(G, H)** Time-resolved curves of single-offset DGE (G) and MTR_asym_ DGE (H) signals, comparing original and corrected data. **(I)** Time-resolved *T_2_* value curves derived from dynamic *T_2_* mapping. **(J-M)** Correlation analysis between *T_2_* values and DGE signals: single-offset DGE vs. *T_2_* for original (J) and corrected (K) data; MTR_asym_ DGE vs. *T_2_* for original (L) and corrected (M) data. Significance levels: 0.001 < **p < 0.01.

The single-offset DGE and MTR_asym_ DGE images of the rat brain parenchyma before and after correction are shown in **Figure 5A**, **5B**, **5D**, **and 5E**. MRS spectra acquired before (blue) and after (red) D-glucose injection demonstrated clear spectral differences (green) within the resonance range of glucose H2-H6 protons (3.0-4.0 ppm; **Figure 5C**). Quantitative analysis revealed that glucose concentration increased from 1.16 ± 0.29 mM pre-injection to 2.33 ± 0.64 mM post-injection, corresponding to an increase of 1.17 ± 0.52 mM (P = 0.005, effect size = 2.357; **Figure S11**), confirming glucose accumulation in the brain parenchyma. In all four experimental groups, both DGE metrics exhibited a gradual increase over time, reaching an approximate steady state around 20 minutes post-injection (**Figure 5G-H**). Concurrently, dynamic *T_2_* mapping (**Figure 5I**) revealed a slow decrease in *T_2_* values within the ROI shown in **Figure 5F**, from 44.3 ± 0.6 ms to 43.0 ± 0.6 ms, consistent with expected physiological changes. Application of the *T_2_* correction led to notable changes in the signal profiles. The peak single-offset DGE signal was significantly reduced from 1.62 ± 0.19% to 1.11 ± 0.16% (P = 0.009), corresponding to an overestimation of glucose concentration by 0.54 ± 0.34 mM (~46%) based on LCModel analysis. Conversely, the MTR_asym_ DGE signal increased from 1.29 ± 0.20% to 1.84 ± 0.18% (P = 0.005), indicating an initial underestimation of 0.35 ± 0.22 mM (~30%) prior to *T_2_* correction. Additional comparisons of fitted uptake parameters are presented in **Figure S12**, and regional comparisons of parenchymal DGE signals, quantified by area under the curve (AUC), are presented in **Figure S13**. To further evaluate the influence of *T_2_* on CEST signal quantification, correlation analyses were performed in the brain parenchyma. For single-offset DGE, the uncorrected signal showed a moderate negative correlation with *T_2_* (R = -0.476, P < 0.001; **Figure 5J**), which was abolished after correction (R = -0.007, P = 0.948; **Figure 5K**). Similarly, MTR_asym_ DGE exhibited a weak negative correlation before correction (R = -0.292, P = 0.0085; **Figure 5L**), which was also eliminated following correction (R = -0.049, P = 0.664; **Figure 5M**). These results confirm that the correction method effectively reduces *T_2_* dependence, thereby improving the robustness of glucose uptake measurements.

**Figure 6:**
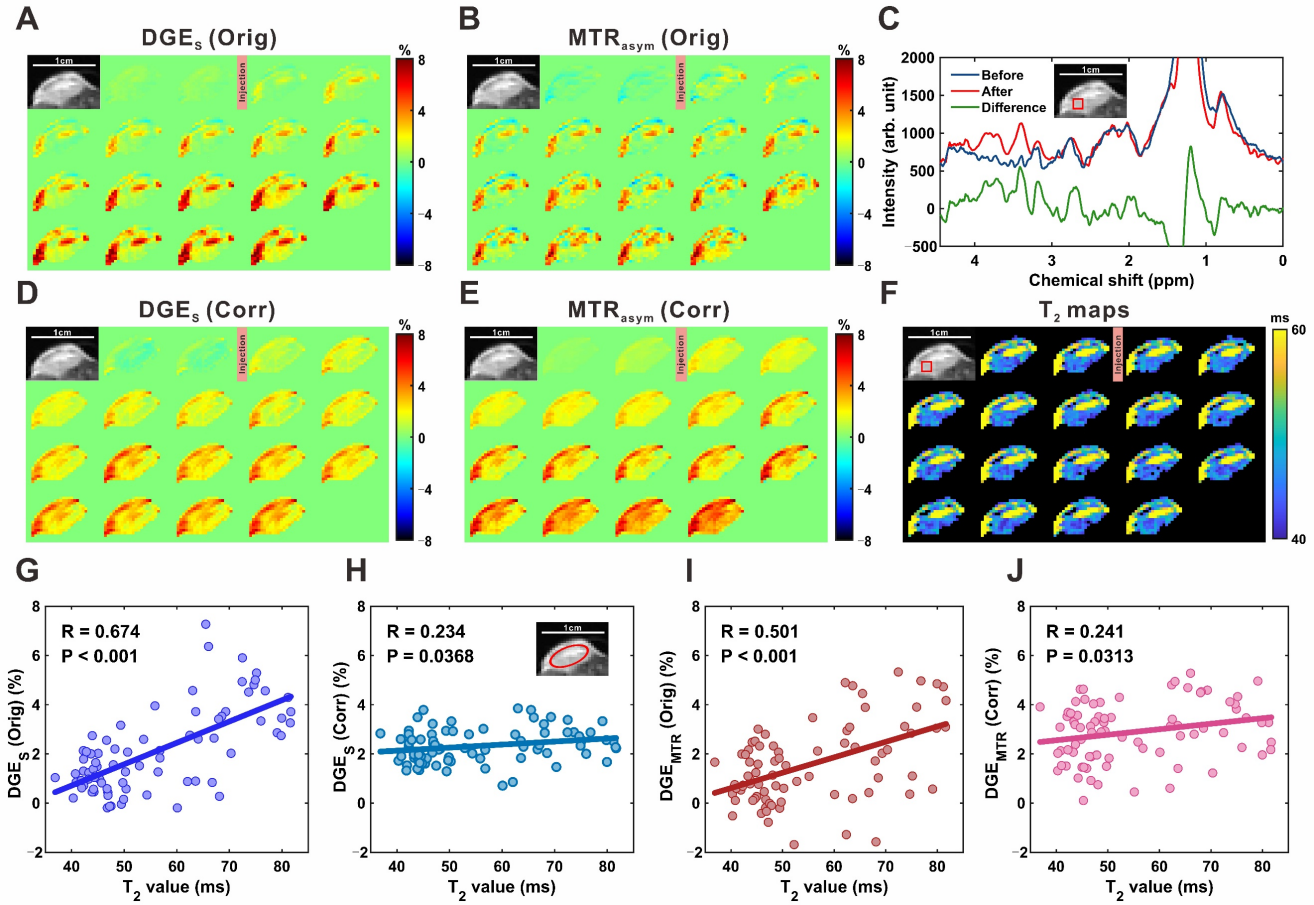
** MP-CEST MRI results of subcutaneous tumor xenograft following D-glucose infusion. (A, D)** Single-offset DGE images before and after glucose infusion, shown for original (A) and *T_2_*-corrected (D) data. **(B, E)** MTR_asym_-based DGE images for original (B) and corrected (E) datasets. **(C)** MRS spectrum indicating glucose-enhanced peaks between 3 and 4 ppm following infusion. **(F)** Dynamic *T_2_* maps before and after glucose infusion, reflecting relaxation changes within the tumor. **(G, H)** Scatter plots showing correlations between single-offset DGE signals and *T_2_* values for original (G) and corrected (H) data. **(I, J)** Correlation plots between MTR_asym_ DGE signals and *T_2_* values for original (I) and corrected (J) data.

**Figure 6A**, **6B**, **6D**, **and 6E** show single-offset DGE and MTR_asym_ DGE images of subcutaneous tumors before and after *T_2_* correction. Localized MRS confirmed glucose uptake, with a marked increase in glucose resonance peaks within the 3.0-4.0 ppm range following infusion (**Figure 6C**), consistent with elevated intratumoral glucose levels. Compared to brain parenchyma, tumor DGE signals exhibited a sustained increase over time (**Figure S14**) and significantly higher signal intensities, reflecting enhanced uptake. *T_2_* values concurrently decreased in tumor regions, from 47.5 ± 0.6 ms to 44.0 ± 0.8 ms in the region outlined in **Figure 6F**, and from 77.1 ± 3.3 ms to 62.3 ± 2.6 ms in another region shown in the supplementary material (**Figure S15**). Notably, greater *T_2_* reductions coincided with more pronounced DGE signal increases, suggesting a relationship between *T_2_* shortening and elevated glucose accumulation. To quantify this relationship, pixel-wise correlation analyses were performed. For single-offset DGE, the uncorrected signal showed a strong positive correlation with *T_2_* (R = 0.674, P < 0.001; **Figure 6G**), which was significantly reduced following correction (R = 0.234, P = 0.037; **Figure 6H**). Similarly, MTR_asym_ DGE exhibited a moderate pre-correction correlation (R = 0.501, P < 0.001; **Figure 6I**) that was attenuated post-correction (R = 0.241, P = 0.031; **Figure 6J**). These findings demonstrate that the proposed correction strategy accounts for *T_2_*-related contributions in tumor CEST imaging, improving the linearity between DGE signal changes and glucose concentration.

## Discussion

In this study, we present a single-shot multiparametric CEST MRI approach that enables simultaneous acquisition of saturation-weighted PD and *T_2_* maps, along with correction of *T_2_* variation-induced signal changes in DGE imaging arising from both *T_2_* decay and spillover effects. Validation in phantom experiments demonstrated high agreement between the estimated PD and *T_2_* maps and reference spin-echo measurements. Importantly, we found that dynamic *T_2_* changes during glucose uptake can bias both single-offset DGE and MTR_asym_ signals, leading to potential overestimation or underestimation of glucose-related contrast. By incorporating *T_2_* calibration into the DGE processing pipeline, these confounding effects were substantially mitigated. The effectiveness of the proposed correction strategy was further confirmed through *in vivo* experiments in both the rat brain and subcutaneous tumor models.

Changes in tissue glucose concentration can alter *T_2_* relaxation through chemical exchange between glucose hydroxyl protons and water protons 21, 43, thereby introducing confounding factors in glucoCEST signal quantification. A key contributing factor is the modulation of spillover in the Z-spectrum, which is mediated by changes in *T_2_* relaxation 44. Specifically, a decrease in *T_2_* broadens the water resonance peak, thereby reducing the frequency selectivity of the saturation pulse and increasing spectral overlap with glucose hydroxyl protons 45, 46. In addition to its effect on spillover, *T_2_* also influences glucoCEST contrast during signal acquisition. Following saturation, the transverse magnetization decays as a function of *T_2_*, and shorter *T_2_* values result in greater signal loss prior to readout 47. This decay can alter the observed CEST contrast, potentially leading to biased estimates of glucose uptake, particularly in regions with short *T_2_* values. A schematic is presented in **Figure S16** to facilitate understanding of how *T_2_* variations affect both spillover during saturation and signal attenuation during acquisition.

Different CEST acquisition schemes and quantification approaches exhibit varying sensitivity to glucose-induced *T_2_* variations. Previous studies have shown that even when using the same pulse sequence, sequence parameters need to be carefully optimized to minimize the confounding effects of *T_2_* variation 48. Some advanced CEST techniques, such as on-resonance variable delay multi-pulse (onVDMP), rely on multiple binomial pulses to generate CEST contrast. While this approach enhances sensitivity to exchangeable protons, its signal formation is inherently more susceptible to *T_2_* effects, as the cumulative magnetization buildup and decay are strongly influenced by transverse relaxation throughout the pulse train. Given this heightened *T_2_* sensitivity, glucose-induced *T_2_* variations may, at least in part, account for the unexpectedly elevated signal intensities reported in previous studies using onVDMP-based acquisitions 49-51. In addition to acquisition schemes, the choice of quantification approach also plays a critical role in determining the sensitivity of glucoCEST measurements to *T_2_* variations. Among the most commonly used methods are the single-offset DGE approach and MTR_asym_-based quantification, both of which differ in their susceptibility to *T_2_*-related confounds. The single-offset DGE method estimates CEST contrast based on signal changes at a single frequency offset, typically near the glucose resonance (e.g., +1.2 ppm). Owing to its single-point sampling, this approach lacks internal mechanisms to separate glucose-specific effects from non-specific background contributions 52. Consequently, the measured DGE signal reflects a combination of true CEST contrast and *T_2_*-dependent spillover, making it particularly vulnerable to *T_2_* variations. In contrast, MTR_asym_ applies an asymmetry analysis by subtracting the signal acquired at the symmetric frequency offset, which substantially eliminates the symmetric spillover contribution 53. However, the resulting CEST contrast remains modulated by *T_2_*-induced changes in background signal intensity. Specifically, variations in *T_2_* alter the amplitude of the background signal, introducing a second-order dependence of MTR_asym_, a phenomenon referred to as the scaled-down effect 30, 31. Therefore, despite its ability to suppress symmetric artifacts, MTR_asym_-based DGE quantification remains sensitive to *T_2_* fluctuations through background-driven modulation.

Glucose-induced *T_2_* variations can affect the sensitivity and specificity of DGE measurements, yet their impact has been largely overlooked in previous studies. To address this issue, we developed two complementary strategies. First, we proposed the use of saturation-weighted PD signals to mitigate the influence of *T_2_* decay during image acquisition. The saturation-weighted PD signal more accurately reflects the saturated magnetization state immediately following the saturation pulse, and is, in theory, more directly related to the intended CEST contrast 22. The feasibility of using saturation-weighted PD signals to generate Z-spectra and quantify glucoCEST effects is demonstrated in **Figure 2**. Second, we introduced a *T_2_* mapping-based correction framework for glucoCEST, in which dynamic *T_2_* maps were used to derive correction models for both single-offset DGE and MTR_asym_-based quantification. As shown in **Figures 5** and **6**, the proposed correction strategy substantially reduced the correlation between the CEST signal and *T_2_*, effectively minimizing *T_2_*-related confounds in both single-offset and MTR_asym_-based DGE quantification. This decoupling allows for a more accurate assessment of glucose uptake independent of relaxation effects.

The single-shot MP-CEST sequence, based on SPEN MRI, allows simultaneous acquisition of CEST contrast, *T_2_*, and PD maps within a single scan without extending scan time or increasing protocol complexity. SPEN MRI is an ultrafast imaging technique with acquisition speed comparable to EPI, but offers superior resistance to B_0_ inhomogeneities 28, 54. Previous studies have combined SPEN with CEST to improve robustness against B_0_ inhomogeneities in fast imaging settings 55. Recent studies, along with our previous work, have demonstrated that the quadratic phase encoding and spatially selective decoding intrinsic to SPEN MRI can be leveraged for ultrafast quantification of both *T_2_* and T_2_^*^ 26. Here, SPEN MRI is incorporated into the CEST framework to enable ultrafast, multiparametric imaging within a single acquisition, offering several practical advantages. First, conventional CEST protocols typically require additional T_1_ and *T_2_* mapping sequences for quantification or correction purposes, which increases total scan time and reduces temporal resolution 56. This limitation is particularly problematic in DGE imaging, where separate acquisitions may introduce temporal misalignment, potentially leading to bias due to interleaved or non-synchronized parameter estimation. Second, inconsistencies in acquisition sequences and imaging parameters between conventional CEST and T_1_/*T_2_* mapping can lead to spatial misregistration, which may compromise the accuracy of voxel-wise quantification and reduce the reliability of longitudinal assessments. In contrast, the MP-CEST approach provides inherently co-registered *T_2_*, PD, and CEST maps from a single acquisition, ensuring spatial consistency across parameters 57.

Beyond *T_2_* correction, *T_2_* mapping itself provides complementary physiological and anatomical information that enhances the value of glucoCEST imaging. Specifically, dynamic *T_2_* changes can serve as an independent marker of tissue response to glucose administration, reflecting alterations in the relaxation time of water as well as microenvironmental factors such as edema, cellularity, and water content shifts 21. These *T_2_* variations may indirectly localize glucose uptake, particularly in regions where CEST contrast is weak or ambiguous. In addition, baseline *T_2_* maps offer structural context that aids image interpretation, facilitating the differentiation of tissue types, lesion characterization, and discrimination between necrotic and viable tumor regions 22, 58. By combining *T_2_* mapping with glucoCEST, the resulting multiparametric framework improves both quantitative robustness and physiological interpretability, enabling a more comprehensive assessment of tissue status.

Beyond its technical advancements, the proposed MP-CEST approach shows great potential for molecular therapy monitoring. By using D-glucose, a clinically approved and metabolically active probe, MP-CEST enables noninvasive assessment of tissue metabolism with improved specificity through correction of *T_2_*-related confounding effects. Altered glucose uptake is a key feature of many diseases, including gliomas, neurodegenerative disorders, and immune-responsive tumors 59-61. In this context, changes in corrected DGE signals may provide early biomarkers of therapeutic response, capturing alterations in glycolytic activity or perfusion before anatomical changes become apparent. Compared to radioactive PET, MP-CEST is more suitable for longitudinal studies, as it avoids ionizing radiation and permits repeated imaging sessions over time 62. Moreover, the simultaneous acquisition of quantitative *T_2_* maps offers complementary information related to tissue water content, cellularity, and microstructural integrity 63, all of which are relevant to treatment efficacy. These features support the utility of MP-CEST as a valuable imaging technique for tracking molecular-targeted therapies, evaluating metabolic interventions, and assessing drug responses across preclinical studies and potential clinical applications.

While the proposed approach demonstrates significant advantages in glucoCEST quantification, several methodological considerations should be noted. First, although the proposed MP-CEST method enables the simultaneous acquisition of multiple parametric maps within a single shot, the optimization of imaging parameters, particularly the number and timing of echo trains, requires careful consideration. Short echo trains may compromise spatial resolution, introducing partial volume effects that can degrade quantification accuracy. Conversely, longer echo trains improve *T_2_* sensitivity but suffer from lower SNR, especially at extended echo times. Parameter selection should therefore be tailored to the expected *T_2_* range of the target tissue. In future work, spatial resolution may be enhanced using deep learning-based super-resolution techniques 64-66, while the intrinsic redundancy across multi-echo images may be leveraged by advanced denoising strategies to further improve image quality and glucoCEST accuracy 67, 68. Second, the *T_2_* correction implemented in this study relies on a calibration curve derived from numerical simulations that model the relationship between CEST signal, glucose concentration, and *T_2_* relaxation time under defined experimental assumptions. As the calibration is inherently dependent on parameters such as magnetic field strength, saturation scheme, and tissue properties, its applicability must be adjusted for different acquisition settings. In this work, simulation parameters were selected based on physiologically relevant literature values 69. However, future implementations may require recalibration to accommodate changes in field strength or tissue type, such as when translating from preclinical to clinical MRI. Careful adaptation of these parameters is essential to maintain the accuracy, robustness, and transferability of the *T_2_* correction strategy across diverse imaging contexts.

Future extensions of this approach may involve integrating glucoCEST with additional imaging contrasts, such as diffusion-weighted imaging, which provides complementary information on perfusion, cellularity, and tissue microstructure 25. Multiparametric integration has the potential to improve physiological specificity and enable a more comprehensive characterization of metabolic alterations across a range of healthy and pathological conditions. Beyond glucose imaging, the proposed acquisition and correction framework may also be adapted to other CEST techniques, such as CrCEST 66, 70, to monitor phosphocreatine-to-creatine conversion during muscle activity. As exercise-induced metabolic shifts are often accompanied by changes in *T_2_*, incorporating dynamic *T_2_* mapping could further enhance the accuracy of CEST signal interpretation in both neurological and neuromuscular applications.

## Conclusion

In this study, we introduced a single-shot multiparametric CEST MRI sequence based on SPEN, enabling the concurrent acquisition of CEST contrast, *T_2_*, and proton density maps within a single scan. This framework incorporates two complementary correction strategies designed to mitigate *T_2_*-related confounds affecting both the saturation and acquisition phases. Validation in phantom and *in vivo* models demonstrated improved DGE quantification and enhanced correspondence between corrected signals and glucose concentration dynamics. These findings establish a robust and generalizable framework for glucoCEST imaging, supporting a more specific assessment of *in vivo* glucose uptake by separating *T_2_* effects from DGE contrast.

## Supplementary Material

Supplementary methods and figures.

## Abbreviations

CEST: chemical exchange saturation transfer
DGE: dynamic glucose-enhanced
SPEN: spatiotemporal encoding
PD: proton density
FDG-PET: [18F]-fluorodeoxyglucose positron emission tomography
MRS: magnetic resonance spectroscopy
glucoCEST: glucose CEST
MP-CEST: multiparametric CEST
PBS: phosphate-buffered saline
PRESS: point-resolved spectroscopy
OVS: outer volume suppression
VAPOR: variable power RF pulses and optimized relaxation delays
ROI: region of interest
