# Supplementary material for: Single-shot multiparametric MRI for separating T2 effects from dynamic glucose-enhanced contrast
Source: Theranostics. 2025 Aug 30;15(18):9678–94. doi: 10.7150/thno.116483 (PMC12486256; doi:10.7150/thno.116483)
Supplement: Supplementary file 1 — Supplementary methods and figures. [file thnov15p9678s1.pdf]

Supporting Information

Single-shot multiparametric MRI for separating T_2 effects from dynamic glucose-enhanced contrast

Junxian Jin[1], Haizhen Ding[2], Zhekai Chen[1], Yuan Huang[1], Hongmin Chen[2], Zhong Chen[1*], Lin Chen[1*]

1 Department of Electronic Science, Fujian Provincial Key Laboratory of Plasma and Magnetic Resonance, School of Electronic Science and Engineering, National Model Microelectronics College, Xiamen University, Xiamen, China

2 State Key Laboratory of Vaccines for Infectious Diseases, Xiang An Biomedicine Laboratory, National Innovation Platform for Industry-Education Integration in Vaccine Research, School of Public Health, Xiamen University, Xiamen, China

*Corresponding Author:

Lin Chen

Xiamen University, Xiamen, China

E-mail: chenlin21@xmu.edu.cn

Zhong Chen

Xiamen University, Xiamen, China

E-mail: chenz@xmu.edu.cn

Supplementary Section 1. Data processing workflow

The corresponding workflow for generating the T_2 and PD maps is illustrated in **Figure S1**. A one-dimensional Fourier transform was first applied along the readout dimension. Even and odd echo train datasets were then separated and individually processed using a super-resolution transformation to correct for blurring caused by parabolic phase profiles [1]. A self-referenced correction step was incorporated to mitigate gradient system imperfections and motion-related artifacts [2]. To ensure consistent image orientation, images from even echoes were flipped due to the opposite spatial rasterization directions along the SPEN dimension. Effective echo times (TE_{eff}) were calculated based on the spatial position along the SPEN axis. Finally, voxel-wise mono-exponential fitting was performed using Eq. 4 to derive quantitative T_2 and PD maps.

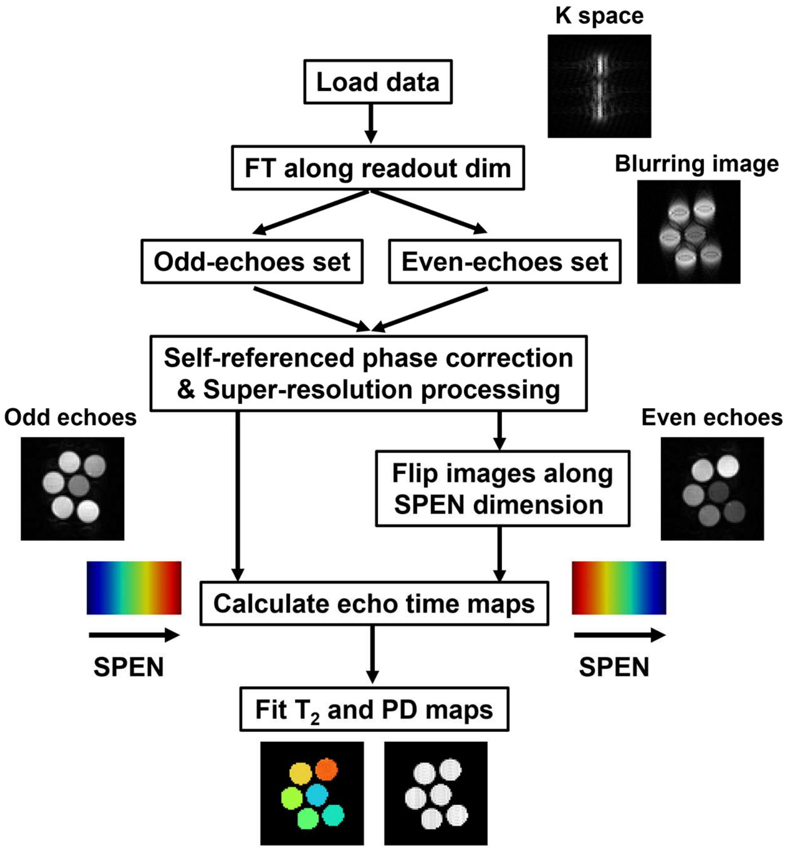

Figure S1. Data processing workflow for generating T_2 and PD maps from MP-CEST imaging.

A representative fitting result from one of the phantoms is shown in **Figure S2**. The fitted curve demonstrated satisfactory agreement with the acquired multi-echo data, yielding T_2 and normalized PD fitting errors within ±1.5 ms and 2%, respectively (**Figure S2A and Table S4**). Residual analysis further validated the reliability of the fitting, with deviations between the measured and fitted signal intensities remaining below 2.5% across all data points (**Figure S2B**).

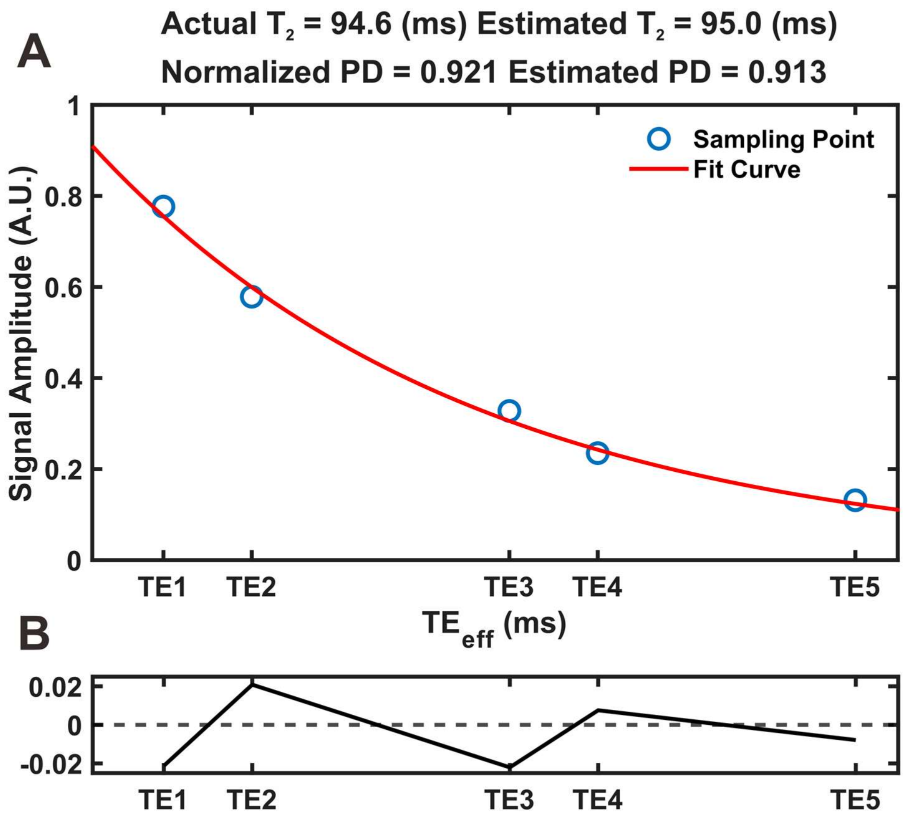

Figure S2. Representative results of T_2 and PD fitting and residual analysis. **(A)** Multi-echo signal intensities (dots) and the corresponding voxel-wise mono-exponential fitting curve (line) from one phantom voxel. **(B)** Residuals between the measured and fitted signals, demonstrating fitting accuracy within 2.5%.

Supplementary Section 2. Validation of model robustness and generalizability

Details regarding the calibration parameters a, b, c, and k are provided, including the fitting procedure and the assessment of parameter stability. In addition, we extended the validation of the proposed model by incorporating both numerical simulations and phantom experiments to evaluate its robustness and generalizability.

Table S1. Simulation parameter settings used to generate T_2 correction coefficients based on the Bloch–McConnell model.

Parameters for generating correction parameters			
**Category / Condition**	**Phantom**	**Brain**	**Tumor**
B_0	7 T	7 T	7 T
Saturation power	2 μT	2 μT	2 μT
Saturation length	2 s	2 s	2 s
Z-spectral range	-5 ~ 5 ppm	-5 ~ 5 ppm	-5 ~ 5 ppm
Glucose pool	1.2 ppm	1.2 ppm	1.2 ppm
Glucose exchange rate	1600 Hz	1600 Hz	1600 Hz
Glucose concentration	50 mM	2 mM	2 mM
T_1	2.5 s	1.5 s	1.67 s
T_2	25 ~ 500 ms	25 ~ 70 ms	25 ~ 100 ms
f_{MTC}	——	5%	2%
$T_{2,MTC}$	——	0.0091 ms	0.0091 ms
k_{MTC}	——	40 Hz	40 Hz
MTC offset	——	0 ppm	0 ppm
MTC lineshape	——	Super-Lorentzian	Super-Lorentzian

To validate the relationship between ΔT_2 and background-induced signal changes described in Eqs. 16 and 19, numerical simulations were performed using phantom-matched parameters

(summarized in **Table S1**), with a baseline T_2 of 50 ms and a glucose concentration of 50 mM. The resulting background-induced $\Delta S/S_0$ and ΔMTR_{asym} changes as functions of ΔT_2 are shown in **Figure S3**. As illustrated, $\Delta S/S_0$ exhibits an approximately linear relationship with ΔT_2, while ΔMTR_{asym} demonstrates a quadratic dependence.

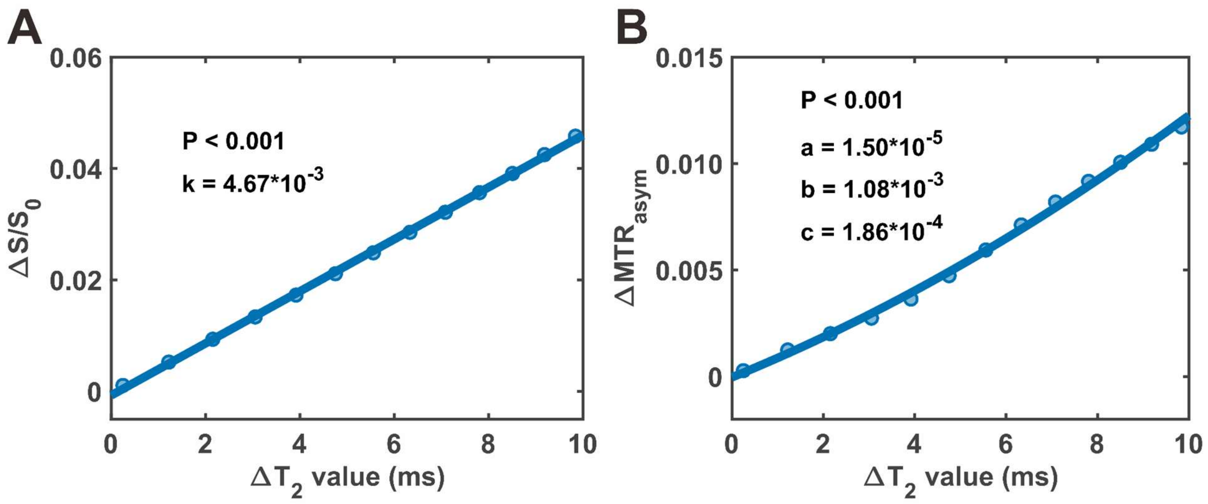

Figure S3. Background-induced signal changes in CEST quantification as a function of ΔT_2. **(A)** $\Delta S/S_0$ as a function of ΔT_2, along with the fitted calibration curve. **(B)** ΔMTR_{asym} as a function of ΔT_2 variation, along with the fitted calibration curve.

Effect of ΔT_2 on background-induced signal changes across broader frequency offsets

Figure S4 shows the dependence of background-induced $\Delta S/S_0$ and ΔMTR_{asym} on ΔT_2 at a fixed glucose concentration across different frequency offsets. **Figure S4A–D** shows that $\Delta S/S_0$ exhibits an approximately linear relationship with ΔT_2, while **Figure S4E–H** demonstrates a quadratic dependence of ΔMTR_{asym} on ΔT_2. Notably, although the ΔMTR_{asym} signal decreases as the frequency offset moves farther from the glucose resonance at 1.2 ppm, it still reflects a second-order relationship due to the persistent influence of the scaled-down effect.

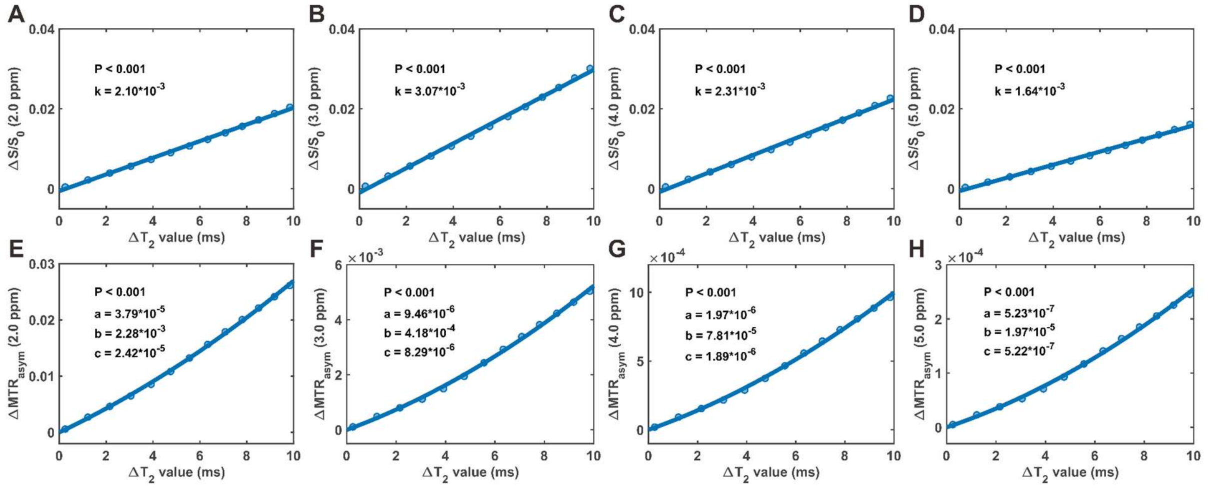

Figure S4. **(A-D)** $\Delta S/S_0$ as a function of ΔT_2, along with the fitted calibration curve at 2 ppm, 3 ppm, 4 ppm, and 5 ppm, respectively. **(E-H)** ΔMTR_{asym} as a function of ΔT_2 variation, along with the fitted calibration curve at 2 ppm, 3 ppm, 4 ppm, and 5 ppm.

To validate the reliability of the correction parameters, additional simulations were conducted to assess the robustness of the T_2 correction model against physiological variations in T1, MTC fraction, and glucose concentration, as shown in **Figure S5**. The simulated background-induced $\Delta S/S_0$ and ΔMTR_{asym} values exhibited excellent resistance to changes in these parameters. Specifically, when T_1 varied from 1200 ms to 1800 ms, $\Delta S/S_0$ showed a relative difference of 3.45% (from 0.030 ± 0.0031 to 0.028 ± 0.0034), and ΔMTR_{asym} varied by 3.51% (from 0.0055 ± 0.00094 to 0.0059 ± 0.00079). With an increase in MTC fraction from 1% to 3%, $\Delta S/S_0$ decreased by 8.77% (from 0.031 ± 0.0046 to 0.026 ± 0.0035), and ΔMTR_{asym} decreased by 13.33% (from 0.0068 ± 0.00076 to 0.0052 ± 0.00056). In comparison, varying the glucose concentration from 1 mM to 3 mM had a negligible impact on $\Delta S/S_0$ (both remained at 0.035), while ΔMTR_{asym} increased from 0.0011 ± 0.00017 to 0.0034 ± 0.00062. Although the relative percentage change is larger, the absolute magnitude of ΔMTR_{asym} remains small, and thus is unlikely to compromise the correction accuracy. These findings confirm the robustness and reliability of the proposed T_2 correction model across a physiologically relevant parameter space, underscoring its applicability in dynamic glucose-enhanced imaging.

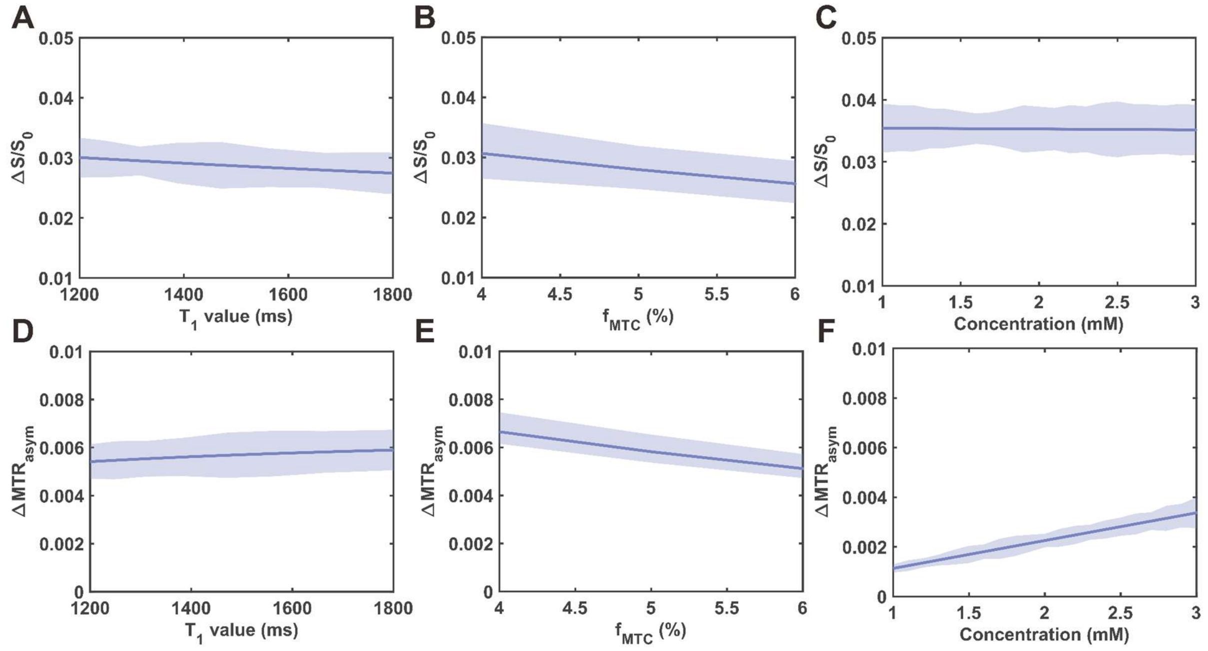

Figure S5. Simulation results showing that the T_2 correction model yields robust performance across a range of T_1 values, MTC fractions, and glucose concentrations. The exchange rate, baseline T_2, and ΔT_2 were set to 1600 Hz, 50 ms, and 10 ms. The remaining simulation parameters are listed in **Table S1**. Background-induced **(A-C)** $\Delta S/S_0$ and **(D–F)** ΔMTR_{asym} as a function of varying (A, D) T_1 values (1200–1800 ms), (B, E) MTC pool fractions (f_{MTC}, 4%–6%), and (C, F) glucose concentrations (1–3 mM).

In vitro validation of T_2 correction

Quantification maps at varying glucose concentrations acquired using MP-CEST are presented in **Figure S6C**. As glucose concentration increased, S/S_0 decreased near the 1.2 ppm offset, whereas MTR_{asym} showed a corresponding increase (**Figure S6A-B**). Quantitative analysis revealed a linear inverse relationship between glucose concentration and S/S_0, while MTR_{asym} exhibited a second-order dependence under physiological conditions (**Figure S6D and S6F**), consistent with simulation results. After T_2 correction, the linear correlation between glucose concentration and S/S_0 was attenuated **(Figure S6E**), indicating effective suppression of T_2-dependent spillover effects. In contrast, MTR_{asym} demonstrated a linear positive correlation with

glucose concentration post-correction (**Figure S6G**), further supporting the correction's ability to minimize T_2-related confounds. It is important to note that T_2 correction requires the definition of a baseline T_2 value, which, in the phantom experiments, was assumed to correspond to the phantom with the highest T_2 (i.e., the lowest glucose concentration).

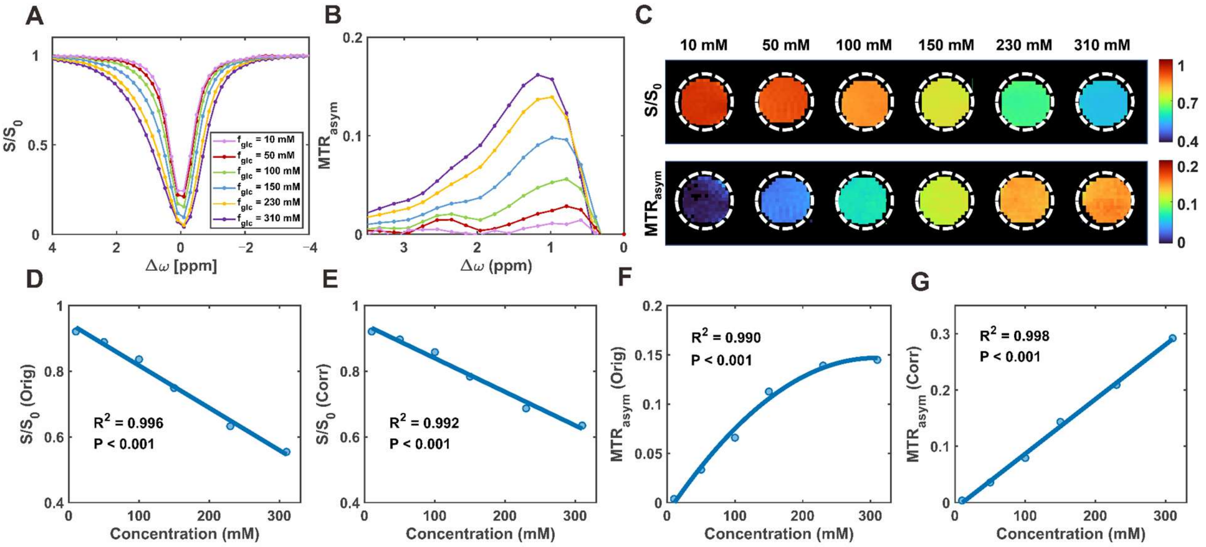

Figure S6. **(A)** Representative Z-spectra acquired from phantoms with different glucose concentrations. **(B)** Corresponding MTR_{asym} curves calculated from the spectra in (A). **(C)** Quantitative maps generated from saturation-weighted PD images prior to T_2 correction. **(D)** Correlation between glucose concentration and normalized signal intensity S/S_0 before T_2 correction. **(E)** Correlation between glucose concentration and normalized signal intensity S/S_0 after T_2 correction. **(F)** Correlation between glucose concentration and MTR_{asym} before T_2 correction. **(G)** Correlation between glucose concentration and MTR_{asym} after T_2 correction.

Supplementary Section 3. Numerical simulation details

The synthetic Z-spectra were generated using Bloch–McConnell equations to simulate the CEST effect under varying T_2 and glucose concentration conditions. All simulations were performed using custom MATLAB scripts, assuming two-pool exchange between water and solute protons. The specific parameters used for the simulations are summarized in Supplementary **Table S2**.

Table S2. Parameter settings for simulating T_2 and glucose concentration effects using the Bloch–McConnell model.

Parameters for generating Z-spectrum	
B_0	7 T
Saturation power	2 μT
Saturation length	2 s
T_1	2.5 s
Z-spectral range	-5 ~ 5 ppm
Glucose pool	1.2 ppm
Glucose exchange rate	1600 Hz
Glucose concentration	0 ~ 500 mM
R_2	10 ~ 36.5 s^{-1}
$r_{2ex, glc}$	0.053 s^{-1} mM^{-1}

Supplementary Section 4. Optimization of echo spacing and number of echoes

In this study, a 5-echo train with an echo spacing of 25 ms was employed in the MP-CEST sequence. For the *in vivo* rat brain and tumor xenograft models, the typical T_2 values range from approximately 30 to 60 ms in healthy brain tissue and extend up to 100 ms in tumor regions. To ensure accurate T_2 quantification across this range, six phantoms with T_2 values spanning 30–100 ms were prepared and imaged using different echo spacings and numbers of echoes. Due to the single-shot nature of the acquisition, echo spacing is typically longer than in multi-shot sequences. An echo spacing of 25 ms was chosen to ensure a balance between multi-echo coverage and adequate in-plane spatial resolution (0.70×0.70 mm^2). Accurate T_2 quantification requires at least three echoes and a total echo time range that adequately covers the expected T_2 distribution. As shown in **Figure S7B**, signal intensity decreases exponentially with increasing TE_{eff} due to T_2 relaxation, resulting in progressively darker images and reduced signal-to-noise ratio (**Figure S7A**). Table S4 summarizes the measured T_2 and PD values from phantom studies using both the MP-CEST and reference spin-echo methods under different echo configurations. Considering the trade-off between fitting accuracy, signal-to-noise ratio, and acquisition efficiency, the 5-echo acquisition was identified as the optimal configuration and was adopted in the final imaging protocol.

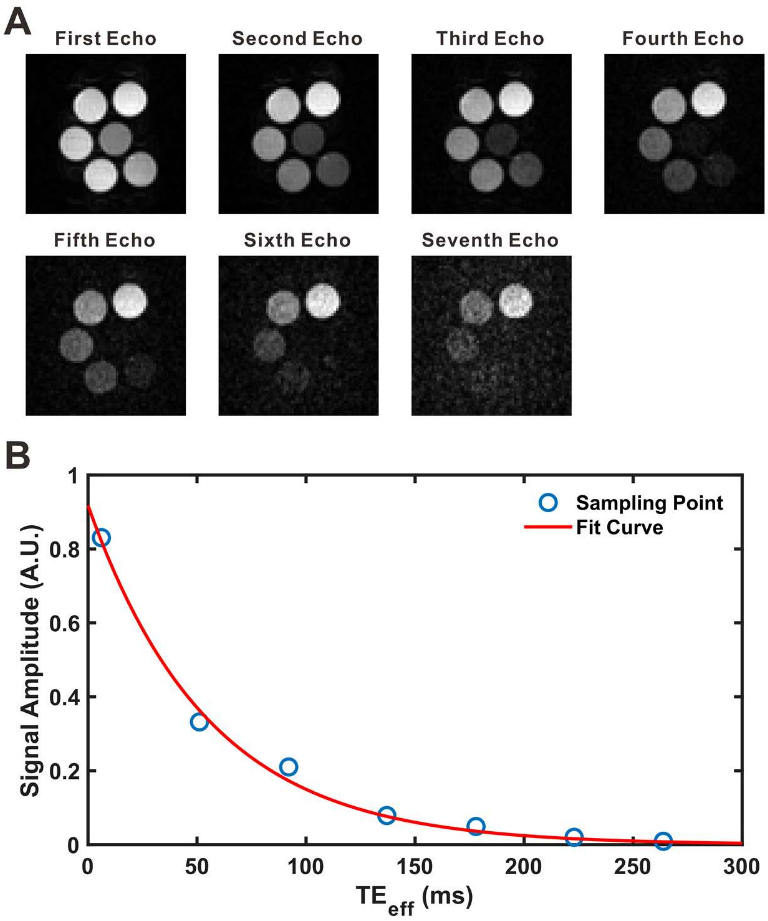

Figure S7. **(A)** Representative reconstructed images obtained using seven echo trains with increasing effective echo times (TEeff), illustrating progressive signal attenuation due to T_2 relaxation. **(B)** Acquired signal intensities (dots) across the echo train and the corresponding mono-exponential fitting curve (line).

Table S3. T_2 and PD measurements in phantoms using different numbers of echoes.

		**MP-CEST**					**Reference**
		**3 shot**	**4 shot**	**5 shot**	**6 shot**	**7 shot**	
**Phantom 1**	**T_2 (ms)**	**33.21 ± 1.33**	33.30 ± 1.35	33.49 ± 1.36	33.48 ± 1.37	33.51 ± 1.38	32.88
	**PD (A.U.)**	**0.900 ± 0.043**	0.894 ± 0.045	0.896 ± 0.046	0.895 ± 0.045	0.896 ± 0.045	0.909
**Phantom 2**	**T_2 (ms)**	39.04 ± 1.26	**39.72 ± 1.26**	40.05 ± 1.30	40.16 ± 1.30	40.22 ± 1.30	39.6
	**PD (A.U.)**	0.883 ± 0.045	0.892 ± 0.032	**0.902 ± 0.042**	0..900 ± 0.041	0.900 ± 0.041	0.910
**Phantom 3**	**T_2 (ms)**	50.09 ± 1.69	50.39 ± 1.56	**50.89 ± 1.55**	51.01 ± 1.53	51.11 ± 1.54	51.00
	**PD (A.U.)**	0.937 ± 0.043	0.932 ± 0.041	**0.927 ± 0.041**	0.927 ± 0.041	0.927 ± 0.041	0.930
**Phantom 4**	**T_2 (ms)**	59.5 ± 3.05	58.86 ± 3.15	**60.10 ± 2.27**	60.35 ± 2.40	60.51 ± 2.25	60.23
	**PD (A.U.)**	0.883 ± 0.071	0.892 ± 0.074	**0.902 ± 0.061**	0..909 ± 0.065	0.901 ± 0.071	0.905
**Phantom 5**	**T_2 (ms)**	74.42 ± 1.76	74.21 ± 1.40	75.11 ± 1.83	**75.72 ± 1.73**	76.45 ± 1.91	75.48
	**PD (A.U.)**	0.938 ± 0.034	0.938 ± 0.037	**0.931 ± 0.038**	0..942 ± 0.042	0.941 ± 0.038	0.931
**Phantom 6**	**T_2 (ms)**	94.74 ± 2.07	92.97 ± 1.45	**94.47 ± 1.29**	94.44 ± 1.47	94.75 ± 1.56	94.61
	**PD (A.U.)**	0.915 ± 0.018	0.918 ± 0.017	**0.919 ± 0.019**	0..917 ± 0.018	0.915 ± 0.021	0.921

Supplementary Section 5. Additional saturation-weighted PD and T_2 mapping results

This section presents additional saturation-weighted PD and T_2 maps acquired using the MP-CEST sequence, which were not included in the main text. The data include both saturated and unsaturated conditions, along with corresponding reference maps for validation. These results are shown in Supplementary **Figure S8**.

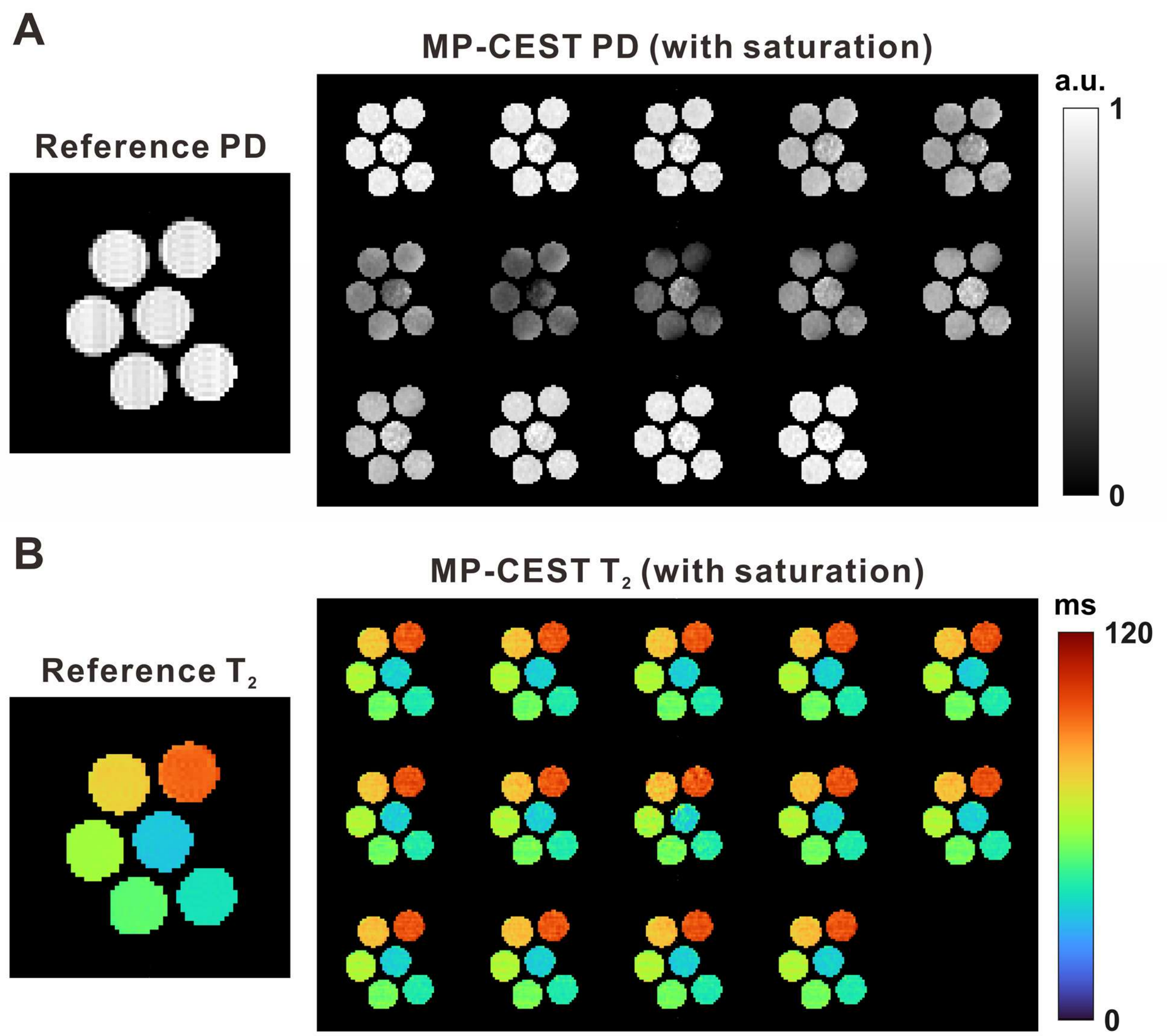

Figure S8. Comparison of quantitative PD and T_2 maps between MP-CEST MRI and reference in vitro measurements. **(A)** PD maps obtained using the MP-CEST method and the reference. **(B)** T_2 maps obtained using the MP-CEST method and the reference.

Supplementary Section 6. Additional Z-spectrum and MTR$_{asym}$ results from phantom experiments

The Z-spectrum and MTR$_{asym}$ plot for the tube containing 10 mM glucose are shown in **Figure S9**.

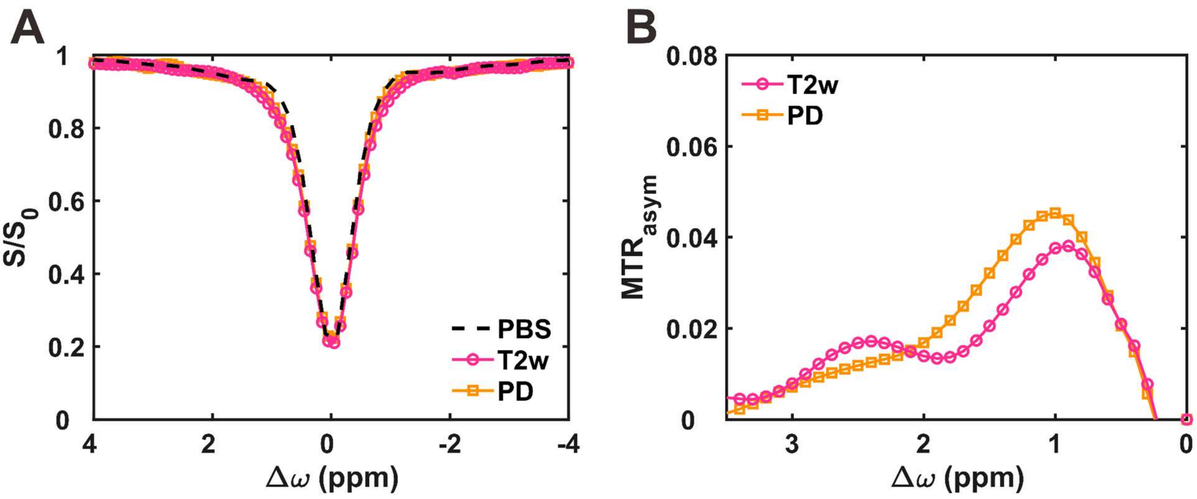

Figure S9. **(A)** Z-spectra derived from T_2-weighted images and saturation-weighted PD images at a glucose concentration of 10 mM. **(B)** Corresponding MTR$_{asym}$ plots calculated from the respective Z-spectra.

Supplementary Section 7. Numerical Validation

Here, a numerical simulation was performed using the parameters in **Table S2** to assess which form of transverse relaxation is more suitable for correction [3]. As shown in **Figure S10**, T_2 demonstrates superior linearity compared with R_2 under the evaluated conditions, with coefficients of determination (R^2) of 0.996 vs. 0.967, 0.999 vs. 0.954, 0.999 vs. 0.944, and 0.991 vs. 0.865 for T_2 and R_2, respectively.

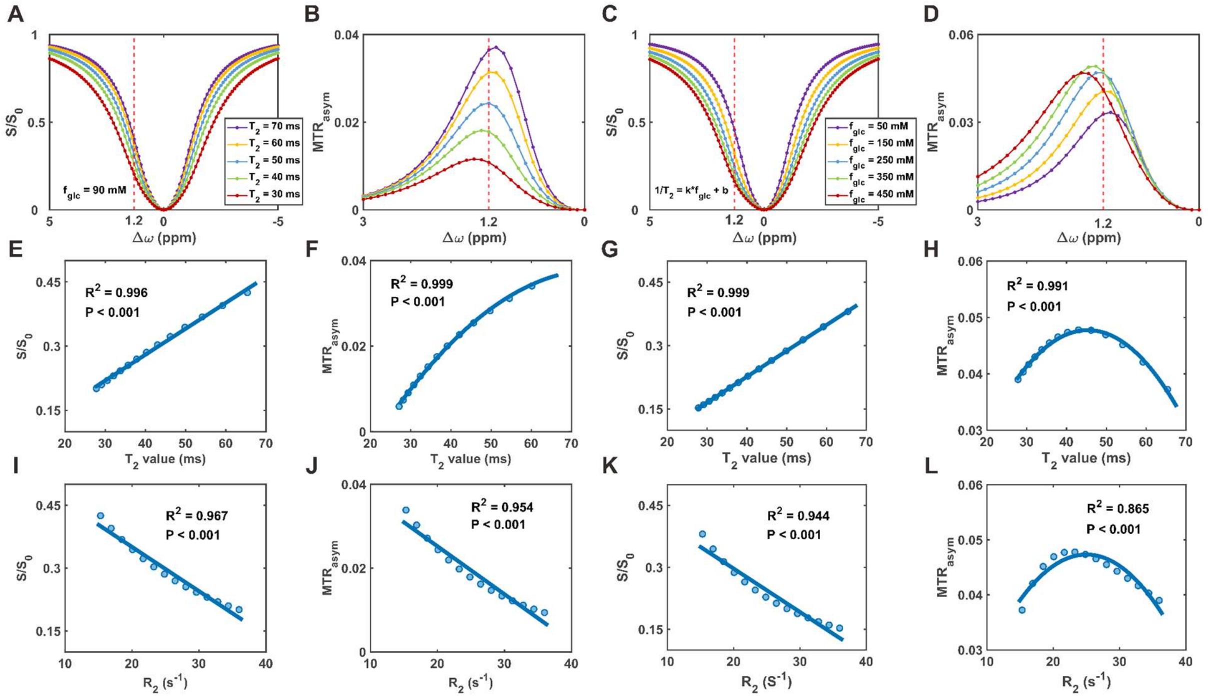

Figure S10. Simulation of glucoCEST signal under varying glucose concentrations and transverse relaxation. (A) Simulated Z-spectra at varying T_2 relaxation times. **(B)** MTR_{asym} curves corresponding to (A). **(C)** Z-spectra under combined variations of T_2 and glucose concentration. **(B)** MTR_{asym} under the same combined conditions. **(E)** Correlation between T_2 and S/S_0. **(F)** Correlation between T_2 and MTR_{asym}. **(G)** Correlation between combined T_2–glucose variations and S/S_0. **(H)** Correlation between combined T_2–glucose variations and $MTR_{asym.}$ **(I)** Correlation between R_2 and S/S_0. **(J)** Correlation between R_2 and MTR_{asym}. **(K)** Correlation between combined R_2–glucose variations and S/S_0. **(L)** Correlation between combined R_2–glucose variations and $MTR_{asym.}$

Supplementary Section 8. LCModel analysis

As shown in **Figure 5C**, MRS spectra acquired before (blue) and after (red) D-glucose injection revealed clear spectral differences (green) within the resonance range corresponding to glucose H2–H6 protons (3.0–4.0 ppm). Based on LCModel analysis, the glucose concentration within the selected ROI increased from 1.16 ± 0.29 mM at baseline to 2.33 ± 0.64 mM after injection, yielding a mean enhancement of 1.17 ± 0.52 mM (P = 0.005, effect size = 2.357), as summarized in **Figure S11**.

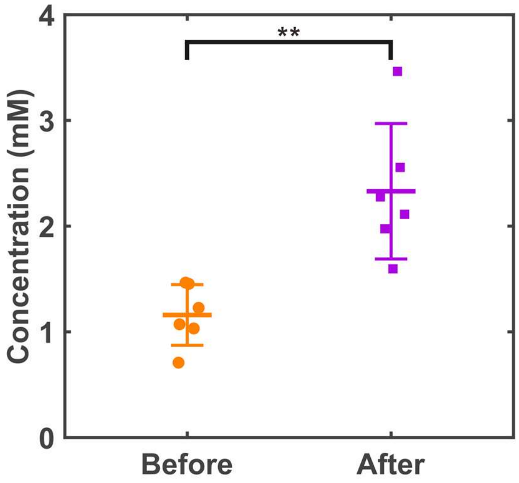

Figure S11. Quantification of glucose concentration before and after D-glucose injection using LCModel. Glucose levels were derived from localized MRS spectra acquired pre- and post-injection, with total creatine (tCr) serving as the internal reference. A significant increase in glucose concentration was observed following D-glucose administration (P = 0.005).

The MP-CEST approach was applied to correct the T_2-induced bias in glucose quantification. As shown in **Figure 5**, the peak single-offset DGE signal was significantly reduced from 1.62 ± 0.19% to 1.11 ± 0.16% after correction (P = 0.009), corresponding to an overestimation of glucose concentration by 0.54 ± 0.34 mM (~46%) based on LCModel analysis. Conversely, the MTR_{asym} DGE signal increased from 1.29 ± 0.20% to 1.84 ± 0.18% (P = 0.005), indicating an initial underestimation of 0.35 ± 0.22 mM (~30%) prior to T_2 correction.

Supplementary Section 9. Glucose uptake analysis

To allow a quantitative comparison of dynamic glucose uptake, time-resolved signal curves in brain parenchyma were fitted using an exponential model:

$$\Delta S(t) = S_{DGE}(1 - \exp(-\mu_{in,DGE} \cdot t)) \quad \text{(S1)}$$

$$\Delta MTR_{asym}(t) = S_{MTR}(1 - \exp(-\mu_{in,MTR} \cdot t)) \quad \text{(S2)}$$

where S_{DGE} and S_{MTR} are the max single-offset DGE and MTR_{asym} DGE signals reached, respectively. $\mu_{in,DGE}$ and $\mu_{in,MTR}$ are the glucose uptake rates by single-offset DGE and MTR_{asym} DGE, respectively. Curve fitting was performed using the built-in nonlinear least-squares fitting function in MATLAB. Statistical analysis was conducted using unpaired, two-tailed Student's t-tests where appropriate. A P value < 0.05 was considered statistically significant.

As shown in **Figure S12**, the fitted S_{DGE} of the original images was significantly larger than that of the corrected (1.62 ± 0.19% versus 1.11 ± 0.16%, P = 0.009), whereas the fitted S_{MTR} of the original images was significantly lower than that of the corrected (1.29 ± 0.20% versus 1.84 ± 0.18%, P = 0.005). In contrast, no significant differences were observed in the uptake rates between original and corrected data: for the rate $\mu_{in,DGE}$, 0.15 ± 0.04 min^{-1} versus 0.16 ± 0.05 min^{-1}; for the rate $\mu_{in,MTR}$, 0.16 ± 0.03 min^{-1} versus 0.15 ± 0.03 min^{-1}.

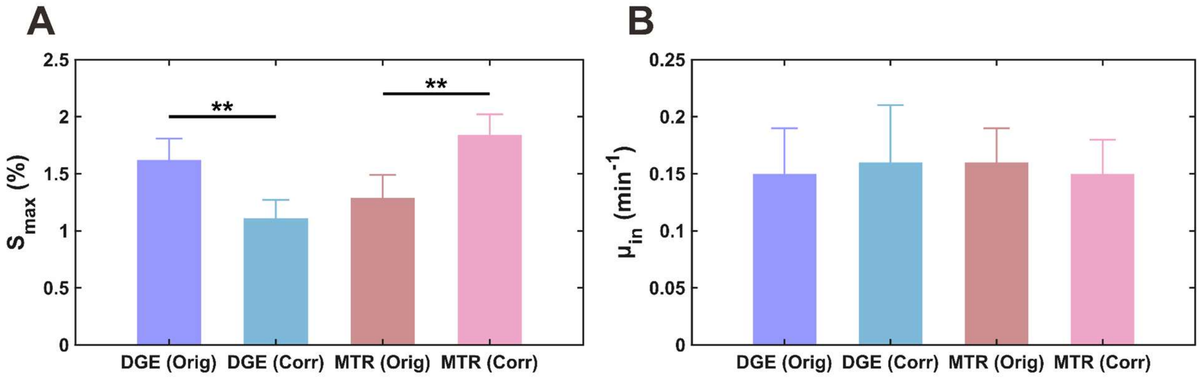

Figure S12. Comparison of model-fitted uptake parameters S_{max} **(A)** and μ_{in} **(B)** for single-offset DGE and MTR_{asym} DGE, analyzed using both the original and the T_2-corrected methods.

Supplementary Section 10. Regional specificity of D-glucose uptake

We incorporated an analysis of region-specific glucose uptake by calculating the area under the curve (AUC) for each ROI, before and after T_2 correction, followed by a comparative assessment across distinct brain regions. The AUC was normalized over a consistent time window to quantitatively assess the single-offset DGE signal differences:

$$AUC = \frac{\sum_{n=1}^{N} DGE_S(n)}{N} \tag{S3}$$

A regional comparison of parenchymal DGE signals is presented in **Figure S13**, where ROIs were selected from the cerebral cortex (CX, purple), hippocampus (HC, green), thalamus (TH, pink), and hypothalamus (HY, red) as shown in **Figure S13A**. To evaluate regional glucose uptake, the AUC was calculated from 2 to 35 minutes post-injection (**Figure S13B**). The results reveal a region-specific pattern of glucose uptake. Before T_2 correction, the hypothalamus exhibited significantly higher uptake than the thalamus (1.41 ± 0.14% vs. 1.22 ± 0.15%, $P = 0.046$), hippocampus (1.41 ± 0.14% vs. 0.96 ± 0.09%, $P < 0.001$), and cortex (1.41 ± 0.14% vs. 1.19 ± 0.11%, $P = 0.012$), consistent with its established role in metabolic regulation and glucose sensing [4, 5]. In contrast, the hippocampus demonstrated significantly lower uptake than both the thalamus ($P = 0.006$) and the cortex ($P = 0.003$). Following T_2 correction, although the overall AUC values were reduced, the same regional differences remained. These findings support the effectiveness of the proposed correction strategy in reducing T_2-related confounding and improving the regional specificity of glucose uptake assessment.

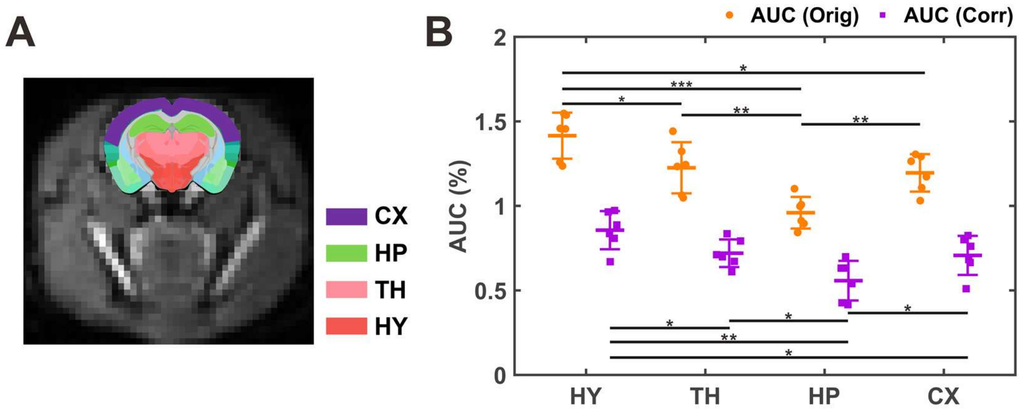

Figure S13. **(A)** Representative brain region segmentation for regional DGE analysis, including the cerebral cortex (CX, purple), hippocampus (HC, green), thalamus (TH, pink), and hypothalamus (HY, red). **(B)** Comparison of the area under the curve (AUC; n = 6) of the single-offset DGE signals before and after T_2 correction across different brain regions, calculated over the 2–35 min window following D-glucose injection.

Supplementary Section 11. Tumor DGE and MTR_{asym} signal analysis

This section presents the dynamic single-offset DGE and MTR_{asym} DGE signals observed in tumor tissue. Unlike in brain parenchyma, both signals continued to increase over time, as illustrated in Supplementary **Figure S14**.

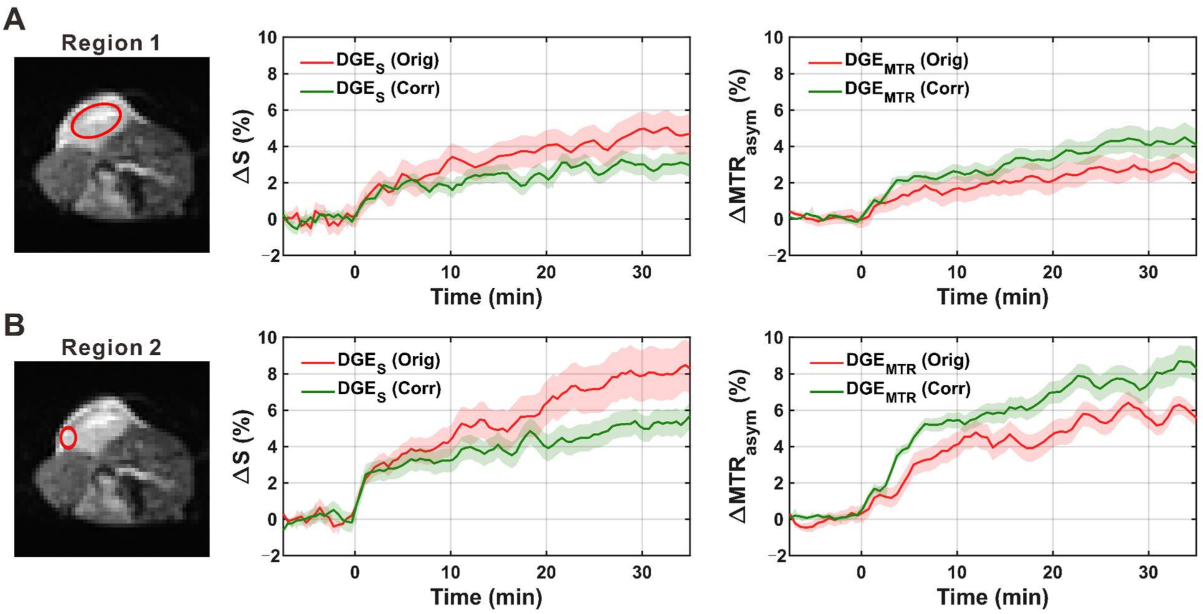

Figure S14. Comparisons of single-offset DGE and MTR_{asym} DGE signals across different tumor regions between original and corrected datasets.

Supplementary Section 12. T_2 Variations following glucose injection in tumor regions

Following glucose infusion, a decline in T_2 relaxation time was observed. In the tumor, T_2 decreased from 47.5 ± 0.6 ms to 44.0 ± 0.8 ms in Region 1 and from 77.1 ± 3.3 ms to 62.3 ± 2.6 ms in Region 2, as shown in Supplementary **Figure S15**. These reductions reflect glucose-induced microenvironmental changes, such as enhanced chemical exchange or restricted water mobility.

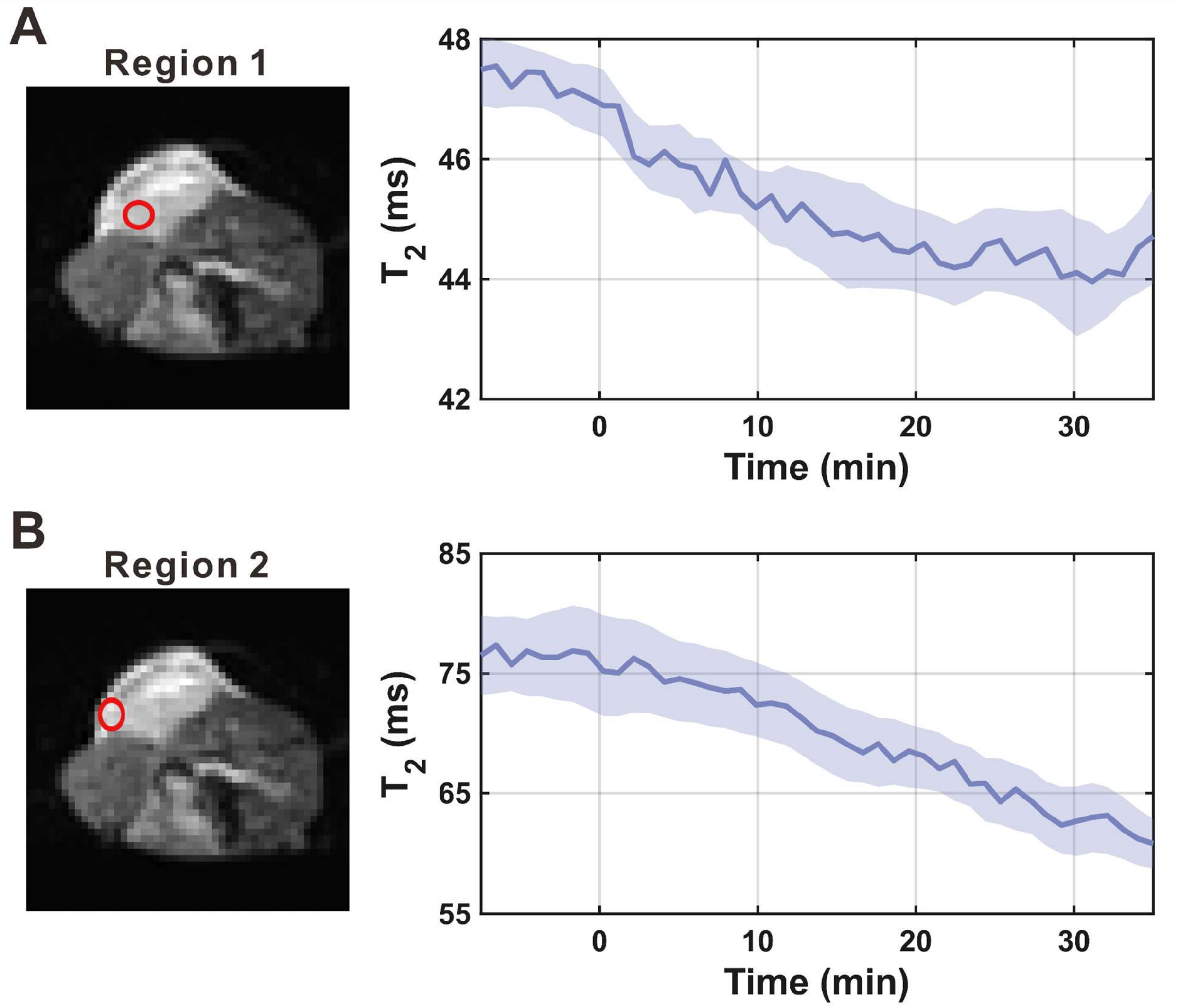

Figure S15. Dynamic T_2 curves in tumor regions following glucose injection. T_2 values decreased over time in both **(A)** Region 1 and **(B)** Region 2, reflecting glucose-induced relaxation changes.

Supplementary Section 13. Influence of T_2 relaxation on Z-magnetization evolution

This section illustrates the effect of T_2 relaxation on the temporal evolution of Z-magnetization during the CEST experiment, as shown in Supplementary **Figure S16**. The figure conceptually demonstrates how changes in T_2 modulate both the saturation transfer process and signal decay during acquisition, thereby introducing confounding factors into glucoCEST quantification.

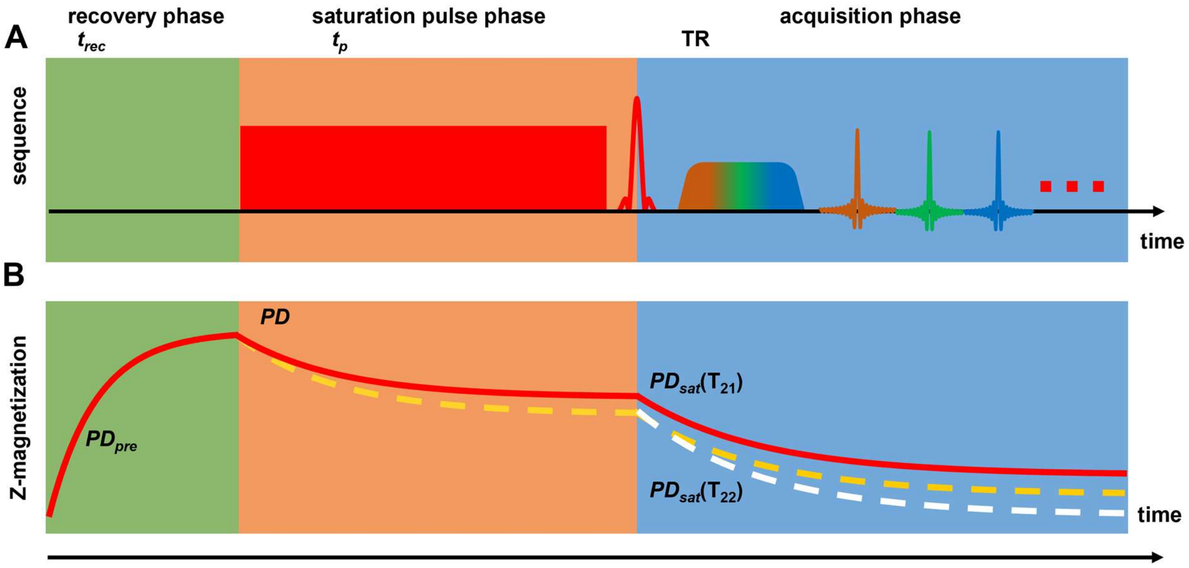

Figure S16. RF pulse sequence diagram **(A)** and corresponding Z-magnetization evolution **(B)** in the single-shot multiparametric CEST MRI experiment. Following the recovery interval, the Z-magnetization of the CEST-labeled acquisition ($PD_{sat}(T_{21})$, dashed yellow line) is lower than that of the reference scan without saturation (noCEST, solid red line). After glucose injection, the tissue T_2 decreases, leading to further attenuation of the saturated signal (PD_{sat} (T_{22}), dashed white line), which falls below PD_{sat} (T_{21}) at the same TE.

References

1. Ben-Eliezer N, Irani M, Frydman L. Super-resolved spatially encoded single-scan 2D MRI. Magn Reson Med. 2010; 63: 1594-600.
2. Li J, Chen L, Cai SH, Cai CB, Zhong JH, Chen Z. Imaging with referenceless distortion correction and flexible regions of interest using single-shot biaxial spatiotemporally encoded MRI. Neuroimage. 2015; 105: 93-111.
3. Yadav NN, Xu JD, Bar-Shir A, Qin Q, Chan KWY, Grgac K, et al. Natural D-glucose as a biodegradable MRI relaxation agent. Magn Reson Med. 2014; 72: 823-8.
4. García-Cáceres C, Quarta C, Varela L, Gao YQ, Gruber T, Legutko B, et al. Astrocytic insulin signaling couples brain glucose uptake with nutrient availability. Cell. 2016; 166: 867-80.
5. Goutal S, Lancien M, Rivier F, Tournier N, Vaillend C. Brain glucose metabolism as a neuronal substrate of the abnormal behavioral response to stress in the mdx mouse, a model of Duchenne muscular dystrophy. Neurobiol Dis. 2025; 204: 106771.
